# Decoupling peptide binding from T cell receptor recognition with engineered chimeric MHC-I molecules

**DOI:** 10.3389/fimmu.2023.1116906

**Published:** 2023-01-25

**Authors:** Georgia F. Papadaki, Omar Ani, Tyler J. Florio, Michael C. Young, Julia N. Danon, Yi Sun, Devin Dersh, Nikolaos G. Sgourakis

**Affiliations:** ^1^ Center for Computational and Genomic Medicine, Department of Pathology and Laboratory Medicine, The Children’s Hospital of Philadelphia, Philadelphia, PA, United States; ^2^ Department of Biochemistry and Biophysics, Perelman School of Medicine, University of Pennsylvania, Philadelphia, PA, United States; ^3^ Department of Radiation Oncology, Perelman School of Medicine, University of Pennsylvania, Philadelphia, PA, United States

**Keywords:** major histocompatibility complex (MHC), antigen presentation, chimeric molecules, T cell receptors, structural immunology, cancer immunotherapy

## Abstract

Major Histocompatibility Complex class I (MHC-I) molecules display self, viral or aberrant epitopic peptides to T cell receptors (TCRs), which employ interactions between complementarity-determining regions with both peptide and MHC-I heavy chain ‘framework’ residues to recognize specific Human Leucocyte Antigens (HLAs). The highly polymorphic nature of the HLA peptide-binding groove suggests a malleability of interactions within a common structural scaffold. Here, using structural data from peptide:MHC-I and pMHC:TCR structures, we first identify residues important for peptide and/or TCR binding. We then outline a fixed-backbone computational design approach for engineering synthetic molecules that combine peptide binding and TCR recognition surfaces from existing HLA allotypes. X-ray crystallography demonstrates that chimeric molecules bridging divergent HLA alleles can bind selected peptide antigens in a specified backbone conformation. Finally, *in vitro* tetramer staining and biophysical binding experiments using chimeric pMHC-I molecules presenting established antigens further demonstrate the requirement of TCR recognition on interactions with HLA framework residues, as opposed to interactions with peptide-centric Chimeric Antigen Receptors (CARs). Our results underscore a novel, structure-guided platform for developing synthetic HLA molecules with desired properties as screening probes for peptide-centric interactions with TCRs and other therapeutic modalities.

## Introduction

The class I proteins of the Major Histocompatibility Complex (MHC-I) present epitopic peptide antigens on the cell surface, thereby enabling immune surveillance of the intracellular proteome by CD8+ T cells and Natural Killer cells (1–5). Under physiological conditions, peptide:MHC (pMHC-I) molecules are assembled in the endoplasmic reticulum (ER) and are trafficked to the cell surface to present a pool of millions of different peptides derived from either host (self-peptides) or aberrant proteins, including viral factors and dysregulated oncoproteins (non-self-peptides) (2). The human MHC-I molecules, referred to as Human Leukocyte Antigens (HLAs), are among the most polymorphic genes with over 35,000 different allotypes reported in the human genome and are classified into the *HLA-A*, *-B*, and *-C* subfamilies (6–10). Several studies have proposed that the vast HLA diversity and extended peptide binding repertoire was driven by evolutionary pressures to adapt in pathogen-rich environments (11–14). Nonetheless, HLAs are structurally conserved with a variable heavy chain, an invariant light chain (β_2_-microglobulin, β_2_m), and a bound peptide typically ranging between 8-15 amino acids in length (15–18). The heavy chain is comprised of three domains, the α_1_ and α_2_ helices define the peptide binding groove in the MHC-I structure, while α_3_ stabilizes the molecule by creating an extensive binding interface with β_2_m. The peptide-binding groove consists of several adjacent ‘pockets’ referred to as A-F, and polymorphisms within the groove govern the respective antigen repertoire of different HLA allotypes, and induce specific peptide conformations (17, 19). While in most HLA allotypes, such as the common HLA-A*02:01 allele, the B- and F-pockets are the primary sites of stabilizing interactions with two specific peptide anchor residues at positions 2 (P2) and 9 (P9), respectively, several allotypes exhibit different anchor residues (20, 21). These variations across different HLA allotypes enable immune surveillance of diverse peptide repertoires at the population level, thus ensuring species adaptability to emerging pathogens (22).

The ability of T cells to recognize epitopic peptides in the context of specific MHC molecules is known as MHC restriction, and two hypotheses have been proposed to explain this phenomenon. The clonal selection theory poses that only TCRs binding specific MHCs will survive thymic selection (23), whereas the germline hypothesis supports that TCRs co-evolved for inherent reactivity to their MHC counterparts (24). However, experimental data for and against both models suggest that they are not mutually exclusive, and can be interpreted by a combined hypothesis (25). Cell-mediated adaptive immune responses depend upon recognition of specific pMHC-I proteins by T cell receptors present in a polyclonal repertoire encompassing 1x10^8^ distinct antigen specificities, leading to stimulation and clonal expansion (26, 27). The association between pMHC-I molecules and TCRs is highly dependent upon interactions with polymorphic residues on the α_1_ and α_2_ helices, as well as with exposed peptide residues. These interactions are mediated by six complementarity-determining regions (CDRs) within the variable domains of the TCR-α and -β chains, which adopt a classical diagonal orientation (25, 28–31). T cells are required to respond to a large number of different epitopic peptides, therefore TCR interactions with their pHLA antigens are characterized by a high degree of cross-reactivity, and inherently low affinity interactions to mitigate the risk of autoimmune responses. A recent study has employed targeted mutagenesis of conserved residues on the α_1_ and α_2_ helices which mediate key germline interactions with TCRs, to enhance recognition by alloreactive T cells while preserving the presentation of peptide antigens in a conserved conformation (32), as a means to break tolerance for specific self-antigens with possible applications in cancer therapy (33). This work provides a rationale for the design of synthetic molecules bridging TCR recognition surfaces with peptide-binding specificities from multiple HLA allotypes as a potential platform for eliciting CD8+ responses against specific tumor-associated antigens. More recently, the advent of peptide-centric, antibody based pMHC engagers as targeting modalities for Chimeric Antigen Receptor (CAR) T cell therapy highlight one additional application of synthetic HLA molecules as probes to screen for and verify allotype-independent recognition of specific antigens with the potential to treat a broader cohort of patients (34). The wide range of peptide-binding specificities covered by the known HLA allotypes is attained through specific combinations of the 33 polymorphic residues which mediate peptide binding (6, 35), suggesting that the peptide-binding groove provides a highly malleable structural scaffold for protein engineering applications aiming to expand naturally occurring T cell repertoires, or to design novel HLA-targeted therapeutics.

Here, we perform an extensive analysis of existing pMHC-I and pMHC-TCR structures to identify key residues that form contacts with peptides and TCRs, respectively. We then outline a systematic, fixed-backbone approach for engineering synthetic MHC-I molecules with desired peptide binding and TCR interface properties. Using the HLA-A*02:01, B*08:01 and B*35:01 alleles as structural scaffolds we generate stable, properly conformed molecules encompassing the peptide-binding specificities of divergent allotypes, including HLA-A*11:01, A*24:02, B*08:01, A*02:01 and C*07:02. We demonstrate that the designed molecules form stable complexes with peptides specific for the desired HLA groove, and adopt an identical conformation compared to their parental, wild-type pMHC-I complexes. Finally, we provide direct evidence that engineered chimeric HLAs presenting disease-related epitopes disrupt interactions with known TCRs but not with peptide-centric CARs, highlighting the importance of HLA framework residues in TCR recognition. Our results underscore a use of chimeric HLAs as screening probes to identify and expand TCR or CAR specificities for distinct peptide antigens, with a minimal reliance on interactions with HLA framework residues. Conversely, in analogy to altered peptide ligands (36, 37), chimeric HLAs provide a rational approach to manipulate interactions between established peptide:HLA antigens and their TCR repertoires in applications aiming to overcome central and peripheral tolerance for eliciting cross-reactive T cell responses against specific self-antigens that are overexpressed in tumor cells, as supported by previous studies (33).

## Materials and methods

### Chimeric MHC-I generation

Chimeric MHC-I molecules were designed using ‘CHaMeleon’, a fixed-backbone approach developed herein. The method requires the structure of an MHC-I allele that binds a desired peptide (groove or template allele), and the sequence of an MHC-I allele with different peptide repertoire and TCR contact surfaces of interest (base allele). The structure of the groove allele was preprocessed to optimize its compatibility with the *Rosetta* software (38). Only the α_1_ and α_2_ helices of the MHC-I heavy chain and the bound peptide were retained, while the conserved α_3_ domain of the heavy chain, the light chain, and all cofactors were removed to reduce the computing time in the subsequent relax protocol. The residues in the structure were renumbered such that the first residue in the structure had residue ID one (**Appendix Script 1**). The peptide binding groove of the template allele was defined as the set of residues within 5 Å of a peptide heavy atom on the processed structure using PyMOL (**Appendix Script 2**). A sequence alignment between the groove MHC-I allele and the base MHC-I allele was performed using EMBOSS Needle pairwise sequence alignment (EMBL-EBI). Starting with the base allele sequence, the chimeric MHC-I sequence was created by substituting every residue in the peptide-binding groove of the base with the corresponding residue of the template allele. To assess the stability and binding affinities of the generated chimeric HLAs, we created and evaluated the structures by threading the chimeric sequence through the preprocessed base allele structure using RosettaCM (**Appendix Script 3**). The threaded structures were then relaxed using the score function ‘REF2015’ in *Rosetta* (**Appendix Scripts 4, 5**). Since we were only interested in the structures that bound the target peptide in the same conformation as the groove allele, the peptide residues were fixed in place using ‘PreventRepackingRLT’. The ‘Fast_Relax Mover’ was used with 3 repeats of the relax protocol allowing both the side chains and backbone of the heavy chain to relax during the simulation. ‘InterfaceAnalyzerMover’ was then used to calculate the binding energy of the peptide to the chimeric MHC-I, after repacking them separately using the ‘pack_seperated’ option. The standard options were used to optimize computational cost while creating realistic relaxed structures (**Appendix Script 4**). The options used in the command line were: ‘-nstruct 3’ to generate three relaxed structures and calculate total and binding energies in each of the triplicates, ‘-no_optH’ to prevent hydrogen placement optimization, ‘flip_HNQ’ to prevent flipping Histidine, Asparagine, and Glutamine, and ‘-use_input_sc’to use the input rotamers as part of the rotamer set explored by the relax algorithm.

### Combinatorial sampling of polymorphic groove residues

An exhaustive assessment of every possible chimeric molecule that could be generated was performed using *Rosetta* software (38). The sequence of the base allele was threaded through the preprocessed structure of the groove allele as described above (**Appendix Script 3**). The threaded structure was then idealized and relaxed using *Rosetta*’s applications with the default options. From three decoy output structures, we used the most stable to introduce each set of mutations on the threaded structure of the base allele using *Rosetta* remodel. A blueprint file was generated for every possible combination of mutations in the polymorphic groove residues between the template and base alleles. For instance, for 9 polymorphic residues between two alleles within 5 Å of the peptide, 2^9^ = 512 blueprint files would be generated and used in conjunction with *Rosetta* remodel to build 512 chimeric-MHC structures. The generated models were refined with a final relax step with a single decoy for each structure and were ranked based on the calculated peptide:MHC binding energy. For the top 2.5% of structures with the lowest energies, we calculated the enrichment score for each polymorphic peptide binding groove position as the ratio of structures among the defined pool, in which a substitution from base to template allele residue was introduced.

### Peptide sequence logo generation

The peptide binding profile of the designed chimeric HLAs was predicted using an in-house method based on NetMHCpan4.0 (39). Briefly, a list of all the experimentally measured peptide epitopes for the MHC class I alleles were extracted from IEDB (7) and were used to predict binding by the chimeric sequences using NetMHCpan4.0. The final sequence logos were generated using Seq2logo (40).

### Recombinant protein expression, refolding, and purification

Plasmid DNA encoding the luminal domain of HLA-A*02:01 and A*24:02 heavy chains, and human β_2_m (β_2_m, light chain) were provided by Dale Long of the NIH Tetramer Core Facility. DNA encoding the HLA-A*11:01-A*02:01, A*11:01-A*02:01^6M^, B*08:01-A*02:01, C*07:02-A*02:01, A*02:01-B*08:01, and A*24:02-B*35:01 chimeric constructs (**Table 1**) was cloned into pET-22b(+) vector using NdeI/BamHI restriction sites (Genscript). For tetramer staining and binding assays, proteins were tagged with the BirA substrate peptide (BSP, LHHILDAQKMVWNHR). The NYE-S1 TCR-α and -β chains were cloned into pET-22b(+) vector with NdeI/BamHI restriction sites (Genscript). DNA plasmids were transformed into *Escherichia coli* BL21(DE3) (New England Biolabs). Proteins were expressed in Luria Broth and inclusion bodies were solubilized using guanidine hydrochloride as previously described (41). pMHC-I complexes were generated by *in vitro* refolding as 200 mg mixtures of heavy chain:light chain at a 1:3 molar ratio and 10 mg of peptide in 1 L of refolding buffer (0.4 M L-Arginine-HCl, 2 mM EDTA, 4.9 mM reduced L-Glutathione, 0.57 mM oxidized L-Glutathione, 100 mM Tris pH 8.0) at 4°C. MHC-I molecules refolded with photolabile peptides were protected from light with aluminum foil. Refolding proceeded for 4 days and the pMHC-I complexes were purified by size-exclusion chromatography (SEC) using a HiLoad 16/600 Superdex 75 pg column at 1 mL/min with 150 mM NaCl, 25 mM Tris buffer, pH 8.0. The luminal domain of the TCR NYE-S1 α/β complex was expressed and purified as previously described (30). The 10LH scFv protein was provided by Myrio Therapeutics (Australia). Protein concentrations were determined using A_280_ measurements on Nanodrop with extinction coefficients estimated by ExPASy ProtParam tool (42).

**Table 1:** Summary of amino acid substitutions introduced in the sequence of a base allele to derive chimeric HLAs.

*Template (Groove) Allele*	Base Allele	Mutations on Base Allele	Resulting Chimeric HLA
A*11:01 (9/18)	A*02:01	G62Q, K66N, H70Q, H74D, V95I, R97I, H114R, Y116D, V152E	HLA-A*11:01-A*02:01
A*11:01 (6/18)		H70Q, H74D, V95I, R97I, H114R, Y116D	HLA-A*11:01^6M^-A*02:01
C*07:02 (14/35)		F9D, A24S, G62R, V67Y, H70Q, T73A, D77S, T80N, V95L, Y99S, H114D, Y116S, W147L, V152A	HLA-C*07:02-A*02:01
B*08:01 (18/35)		F9D, A24S, G62R, E63N, K66I, V67F, A69T, H70N, H74D, V76E, D77S, T80N, V95L, R97S, H114N, Y116N, T142I, L156D	HLA-B*08:01-A*02:01
A*02:01 (11/35)	B*08:01	D9F, E45M, N63E, I66K, F67V, N70H, D74H, S77D, S97R, N114H, D156L	HLA-A*02:01-B*08:01
A*24:02 (16/38)	B*35:01	Y9S, T45M, N63E, I66K, F67V, N70H, Y74D, S77N, L81A, I95L, R97M, Y99F, D114H, S116Y, L156Q, W167G	HLA-A*24:02-B*35:01

The number of amino acid substitutions introduced in the sequence of the base allele, versus the total number of polymorphic residues between the template (groove) and base alleles, are shown in brackets.

### Peptides

A full list of the peptides used in this study and their abbreviations is shown in **Supplementary Table 1**. All peptide sequences are given as standard single-letter codes and were purchased from Genscript, NJ, USA, at >90% purity. The photolabile peptide used was purchased from Biopeptek Inc, PA, USA, using J as 3-amino-3-(2-nitrophenyl)-propionic acid (43). For the peptide solutions, lyophilized peptides were solubilized in distilled water and centrifuged at 14,000 rpm for 15 min. Concentrations were calculated using the respective absorbance and extinction coefficient at 205 nm wavelength.

### Differential scanning fluorimetry

For DSF experiments, samples were prepared at a final concentration of 7 μM in PBS buffer (50 mM NaCl, 20 mM sodium phosphate pH 7.2) and mixed with 10X SYPRO Orange dye (ThermoFisher) to a final volume of 20 μL. Samples were then loaded into a MicroAmp Fast 384-well plate and ran in triplicates (n=3) on a QuantStudio™ 5 Real-Time PCR machine with excitation and emission wavelengths set to 470 nm and 569 nm, respectively. Temperature was incrementally increased at a rate of 1°C/min between 25°C and 95°C to measure the thermal stability of the proteins. Data analysis and fitting were performed in GraphPad Prism v9.

### Peptide exchange

Peptide exchange mediated by UV-irradiation was performed by incubating 7 μM of HLA-B*08:01-A*02:01/FLRGRAJGL with 70 μM of the desired peptide in PBS buffer (50 mM NaCl, 20 mM sodium phosphate pH 7.2) for 1 hour at room temperature (RT), followed by UV-irradiation for 1 hour at 365 nm. Samples were centrifuged at 10,000 rpm for 10 minutes at 4°C to remove aggregates. Peptide exchange was determined by performing DSF analysis in triplicates (n=3), as previously described (44).

### X-ray crystallography and structure determination

Purified HLA-A*11:01-A*02:01/HIV-1 RT and HLA-B*08:01-A*02:01/CMV complexes were concentrated to 12.5-15 mg/ml in SEC Buffer (150 mM NaCl, 25 mM Tris buffer, pH 8.0) and used for crystallization in 1:1 ratio of protein-crystallization buffer at 21 °C by sitting drops. Large plate crystals for HLA-A*11:01-A*02:01/HIV-1 RT were obtained in 0.02 M Sodium/Potassium phosphate, 0.1 M BIS-TRIS propane pH 8.5, 18-22% w/v PEG 3350 after 3 days. Small cubic crystals for HLA-B*08:01-A*02:01/CMV were obtained in 0.2 M Sodium fluoride, 0.1 M BIS-TRIS propane pH 8.5, 20-24% w/v PEG 3350 after 2 weeks. All crystals were harvested in crystallization buffer with 27% ethylene glycol using nylon cryo-loops (Hampton Research) and flash frozen in liquid nitrogen. Complete data collection was performed from single crystals under cryogenic conditions at Advanced Proton Source beamlines 19-ID-D and 24-ID-E for HLA-A*11:01-A*02:01/HIV-1 RT and B*08:01-A*02:01/CMV complexes, respectively. Diffraction images were indexed, integrated, and scaled using MOSFLM and HKL3000 in CCP4 Package. Structures were determined by molecular replacement method using Phaser and the previously published structure of HLA-A*02:01 (PDB ID: 5HHN) as a search model. Model building and refinement was performed using COOT and Phenix, respectively. Full data collection and refinement statistics are given in **Table 2**. Crystallographic figures were created using PyMOL.

**Table 2:** Crystallography data collection and refinement statistics for the HLA-A*11:01-A*02:01/HIV-1 RT and B*08:01-A*02:01/CMV chimeras.

Data Collection	A*11:01-A*02:01/HIV-1 RT	B*08:01-A*02:01/CMV
PDB ID	8ERX	8ESH
Beamline	APS 19-ID-D	APS 24-ID-E
Space Group	P _1_ 2_1_ 1	I 2 3
Unit Cell (Å)	56.35 79.32 57.64 90.00 116.10 90.00	147.37 147.37 147.37 90.00 90.00 90.00
Wavelength (Å)	0.979	0.979
Resolution (Å)^1^	2.0 (2.03-2.00)	2.72 (9.01-2.72)
Rsym^2^	0.119 (0.416)	–
<I/σI>^3^	18.6 (3.5)	24.9 (2.2)
CC(1/2)	0.982 (0.859)	0.99 (0.834)
Completeness (%)^4^	99.6 (99.7)	99.9 (99.4)
Redundancy	3.6 (3.5)	17.4 (7.9)
Refinement		
Resolution (Å)	2.07	2.72
R-Factor ^5^	0.192	0.214
Rfree ^6^	0.231	0.259
Protein atoms	3171	3167
Ligands	1	1
Water Molecules	361	35
Unique Reflections	27641	14510
RMSD^7^		
Bonds	0.002	0.109
Angles	0.534	11.57
MolProbity Score (45)	0.79	1.59
Clash Score (45)	0.97	8.49
Percent Ramachandran plot		
Favored, allowed, outlier (%)	(98, 2, 0)	(97, 2, 0)

^1^Statistics for highest resolution bin of reflections in parentheses.

^2^R_sym_ =Σ_h_Σ_j_ | I_hj_-<I_h_> |/Σ_h_Σ_j_I_hj_, where I_hj_ is the intensity of observation j of reflection h and <I_h_> is the mean intensity for multiply recorded reflections.

^3^Intensity signal-to-noise ratio.

^4^Completeness of the unique diffraction data.

^5^R-factor = Σ_h_ | IF_o_I – IF_c_I |/Σ_h_|F_o_|, where F_o_ and F_c_ are the observed and calculated structure factor amplitudes for reflection h.

^6^R_free_ is calculated against a 5% random sampling of the reflections that were removed before structure refinement.

^7^Root mean square deviation of bond lengths and bond angles.

### Phylogenetic analysis

Multiple sequence alignments of the TCR-contact residues from approximately 10 most common allotypes from each subfamily *HLA-A*, *-B*, and *-C*, and of the α_1_ and α_2_ domains between the most similar wild-type alleles with the designed HLA-A*11:01-A*02:01^6M^ chimera were performed using ClustalOmega (46). Alignment files were further processed in ESPript (47). Phylogenetic trees were generated using best-fit models as calculated by MEGA7 (48) and processed in iTOL (49).

### Biotinylation and tetramer formation

Biotinylation of the pMHC-I and soluble 10LH molecules was performed as previously described (50). In brief, BSP-tagged proteins were biotinylated using the BirA biotin-ligase bulk reaction kit (Avidity), according to the manufacturer’s instructions. For the pMHC-I tetramer formation, Streptavidin-PE (Agilent Technologies, Inc.) at 4:1 monomer:streptavidin molar ratio was added to the biotinylated pMHC-I in the dark, every 10 min at room temperature over 10-time intervals.

### Surface plasmon resonance

SPR experiments were conducted in duplicates or triplicates (n=2 or 3) using a BiaCore T200 instrument (Cytiva) in SPR buffer (50 mM NaCl, 20 mM sodium phosphate pH 7.2, 0.1% Tween-20). Approximately 650 resonance units (RU) of biotinylated-A*02:01/NY-ESO-1, A*02:01-B*08:01/NY-ESO-1, or the scFV 10LH were immobilized at 10 µL/min on a streptavidin-coated chip (GE Healthcare). TCR NYE-S1 or A*24:02/PHOX2B, and A*24:02-B*35:01/PHOX2B were captured on the coated surface followed by a wash-out step with buffer at desired concentrations. Samples were injected over the chip at 25°C at a flow rate of 20 µL/min for 60 sec followed by a buffer wash with 180 sec dissociation time and equilibrium data were collected. The SPR sensorgrams, association/dissociation rate constants (*k*_a_, *k*_d_) and equilibrium dissociation constant K_D_ values were analyzed in BiaCore T200 evaluation software (Cytiva) using kinetic analysis settings or fitted using one-site specific binding by GraphPad Prism v9. SPR sensorgrams and saturation curves were prepared in GraphPad Prism v9.

### 1G4 TCR lentivirus production

Lenti-X 293T cells (Takara) were cultured in DMEM (Gibco), 10% FBS (Gibco), and Glutamax (Gibco) and were plated one day before transfection. Cells were transfected at a confluency of 80-90% with TransIT-293 (Mirus) using pMD2.G (Addgene #12259, gift from Didier Trono), psPAX2 (Addgene #12260, gift from Didier Trono), and pSFFV-1G4. Virus-containing media was collected 24- and 48-hours post-transfection, clarified by centrifugation at 500 g for 10 min, and incubated with Lenti-X concentrator (Takara) for at least 24 hours. Virus was pooled and concentrated 50-100x, resuspended in PBS, aliquoted, and stored at -80°C for subsequent T cell infections.

### Primary human T cell tetramer staining

The studies involving human participants were reviewed and approved by the University of Pennsylvania review board. Written informed consent to participate in this study was provided by the participants. Healthy donor T cells were processed by the Human Immunology Core by magnetic separation of CD8+ T cells. Cells were cultured in Advanced RPMI (Gibco), 10% heat inactivated FBS (Gibco), Glutamax (Gibco), penicillin/streptomycin (Gibco), and 10mM HEPES (Quality Biological), supplemented with 300 U/mL recombinant IL-2 (NCI Biological Resources Branch). T cells were maintained at ~1 million cells/mL and were activated with a 1:1 ratio of Dynabeads Human T-Activator CD3/CD28 beads (Gibco) for 48 hours. 24 hours after initial activation, cells were either left untransduced or were transduced with lentivirus expressing the 1G4 TCR. Cells were debeaded by magnetic separation and expanded in the presence of IL-2. Transduction efficiency was determined by staining with an anti-Vβ13.1-APC antibody (Miltenyi Biotec.), typically greater than 50%. Cells were cryopreserved with CryoStor CS10 (StemCell Technologies). Thawed T cells were recovered and regrown in IL-2-containing complete medium for ~3 days prior to staining. Cells were harvested and washed with PBS, 1% BSA, 2 mM EDTA with 5 µg/mL PE-conjugated tetramers and incubated for 25 min at room temperature with mild agitation. After two washes with an RPMI-based buffer containing 1% FBS, cells were resuspended in 1:1000 Sytox Blue diluted in wash buffer to distinguish dead cells. Samples were processed on an LSR Fortessa (BD) and data analyzed by FlowJo v10.8.1.

## Results

### Structural analysis reveals discrete HLA surfaces for peptide binding and TCR recognition

**Figure 1:**
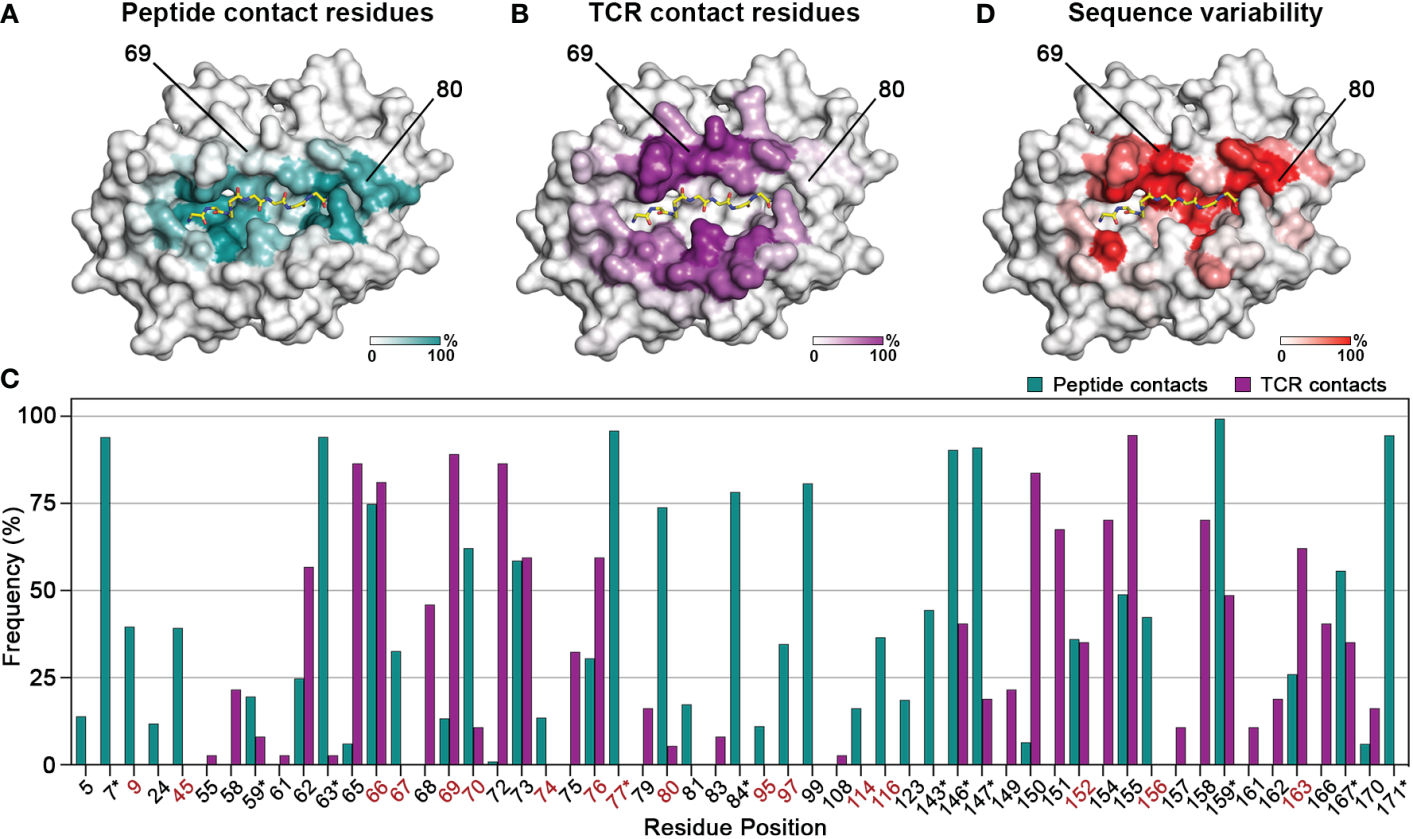
Contiguous molecular surfaces defined by polymorphic HLA residues mediate interactions with peptides, and TCRs. The calculated **(A)** peptide-contact, and **(B)** TCR-contact frequencies of the first 180 amino acids are highlighted in a white (low) and teal or purple (high) gradient, respectively. The structure of HLA A*02:01 (PDB ID: 1S9W) was used as template. **(C)** Bar graph of the peptide- and TCR-contact frequencies for positions with at least one value higher than 2%. Polymorphic positions with a consensus score below 60% are highlighted in red, while residues that form conserved hydrogen bond networks with the peptide main chain are marked with an asterisk (*) (52). **(D)** Sequence variability for each position P plotted as (100 - Consensus score) on the HLA-A*02:01 structure, from a white (low) to red (high) gradient.

We first sought to evaluate the degree of overlap between the residues which mediate interactions with the peptide and T cell receptor complementarity-determining regions, respectively. To do this, we analyzed 384 pMHC-I structures from a curated, in-house database derived from the Protein Data Bank (HLA3DB; https://hla3db.research.chop.edu/) and 36 pMHC-TCR structures from the ATLAS database (51). For each pMHC-I structure, we calculated a peptide-contact frequency as the percent of structures in which each position P of the first 180 amino acids comprising the peptide binding groove was within 4 Å from any peptide heavy atom (**Figure 1A**). Likewise, we calculated a TCR-contact frequency for each P using the available pMHC-TCR structures from the ATLAS database (**Figure 1B**). Based on this analysis, we classified MHC-I positions into three groups: *i)* peptide-only binding (PB) positions that primarily affect peptide binding with a non-zero peptide-contact frequency and a TCR-contact frequency less than 10%, *ii)* TCR-only binding (TB) positions which primarily affect TCR binding with a non-zero TCR-contact frequency and a peptide-contact frequency less than 10%, and *iii)* peptide-TCR binding (PTB) positions that affect both the peptide and TCR binding specificity with peptide- and TCR-contact frequencies greater than 10% (**Figure 1C** and **Supplementary Table 2**). In cases where both frequencies were below 10%, we selected the highest frequency to classify a given residue position as PB or TB. This analysis confirms that the HLA regions that mediate peptide binding show minimal overlap with TCR interaction surfaces.

We next aimed to evaluate the degree of sequence variance among residues belonging to the three identified structural groups, towards understanding whether these positions could be modified to create synthetic molecules with specific binding properties. Therefore, we aligned 2,896 sequences curated from the IMGT/HLA sequence database (53) using as reference the most common allotype HLA-A*02:01, and calculated a consensus score as the frequency of the most common amino acid at each position P. High consensus score implied highly conserved residues whereas low score suggested positions amendable to substitutions without compromising the stability of the pMHC-I complex (**Figure 1D**). For instance, position 80 with a TCR-contact frequency of 5% and a peptide-contact frequency of 74% belongs in the PB category, whereas position 69 with frequencies of 89% and 13%, respectively, is implicated in the formation of more significant contacts with TCRs. Both positions are good targets for designing MHCs with novel peptide or TCR binding profiles, since they have low consensus scores (45% and 42%) and thus are highly polymorphic. On the other hand, nearly all the residues involved in the formation of hydrogen bond networks with the peptide main chain have a consensus score above 90%, implying strictly conserved interactions (52) (**Figure 1C** and **Supplementary Table 2**). Notably, TB residues were overall more conserved, with the lowest consensus score at 67.3% (**Supplementary Table 2**), suggesting that the peptide- and TCR- contact residues followed distinct evolutionary paths to confer adaptability of interactions in the peptide binding groove. Taken together, we demonstrate that results from both structural and sequence analysis can be used to define a set of MHC-I residues that could be altered to modify peptide binding while maintaining the MHC-TCR binding surface intact and *vice versa*.

### Engineering chimeric MHC-I molecules using a structure-guided approach

**Figure 2:**
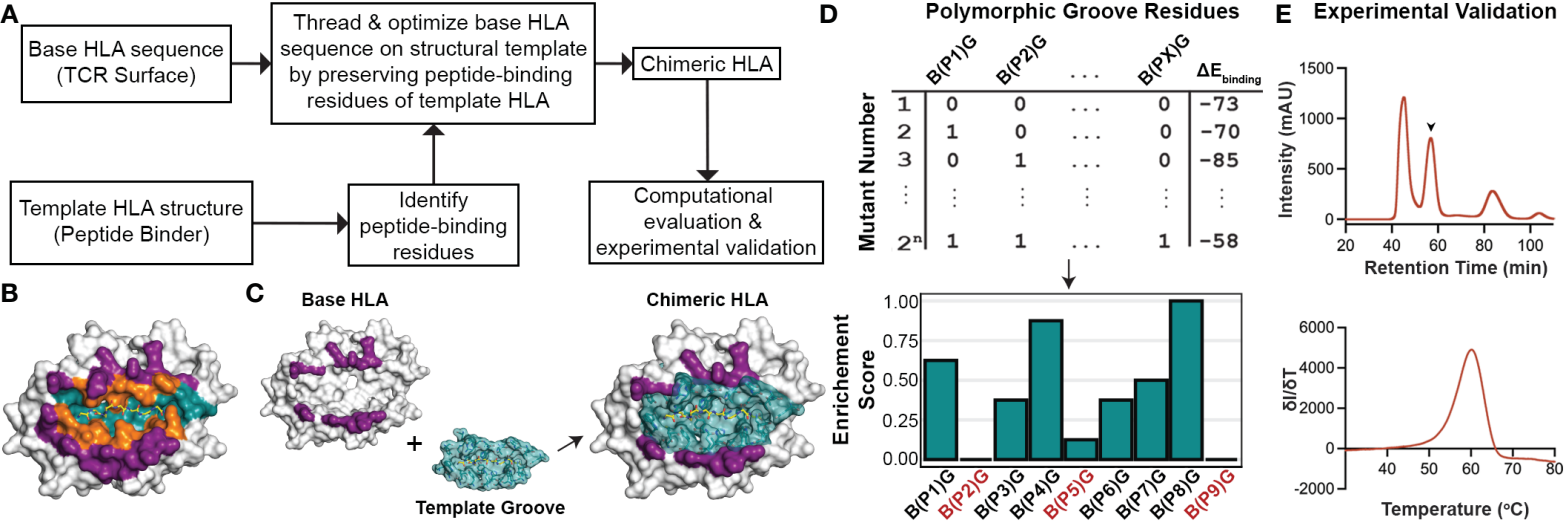
General workflow for generating chimeric HLA molecules using a fixed-backbone, structure-guided approach. **(A)** The general workflow of the CHaMeleon approach to generate chimeric MHC-I molecules. **(B)** The structure of HLA-A*02:01 bound to the peptide SLLMWITQC (PDB ID:1S9W) where the peptide-only (teal), TCR-only (purple) and peptide-TCR-binding (orange) residues are highlighted. **(C)** Grafting the peptide-biding groove of a template onto a base allele to create chimeric molecules. TCR-only positions are highlighted in purple and peptide-only or peptide-TCR-binding residues are highlighted in teal. The structure of HLA-A*02:01 (PDB ID:1S9W) was used as an example. **(D)** Exhaustive combinatorial sampling of groove allele substitutions on the base allele and binding energy calculations was performed to evaluate the chimeric HLA models. The top 2.5% of structures with lowest binding energies were used to calculate Enrichment Scores at each polymorphic position (PX) in the groove, which represents the fraction of chimeric HLAs with a specific mutation from base (B) to groove (G) allele residue. Positions with 0 or very low enrichment scores are highlighted in red. **(E)** Experimental validation of the chimeric pMHC-I by size exclusion chromatography (SEC; top). The protein peak is indicated by the arrow (57.5 min), while the additional peaks correspond to protein aggregates (47 min) and free β_2_m (84 min). Thermal stability of the purified molecules was assessed using differential scanning fluorimetry (DSF; bottom) experiments.

Driven by our sequence and structural analysis, we sought to explore the plasticity of existing HLA structures to accommodate novel peptides using a fixed-backbone design approach. We developed a method called ‘CHaMeleon’, to generate synthetic molecules that combine the peptide binding specificity of one allele (template or groove allele) with the TCR binding surface of another (base allele). Our approach takes as input an existing pHLA template structure and introduces a novel TCR binding surface in three steps: *i)* Generating a threaded model of a base allele sequence using a groove pHLA structural template, *ii)* Model optimization and binding energy analysis to identify the minimal set of mutations necessary to achieve an altered peptide binding specificity, and *iii)* experimental validation of the chimeric MHC-I refolded with the peptide that was observed in the original template structure of the groove allele (**Figure 2A**).

First, we used a 5 Å heavy atom distance threshold to define peptide contacting residues in the structure of a groove HLA with a known antigen, which would be used as a modeling template (**Figure 2B**). Next, we identified polymorphic residues which differ between the sequences of the groove and base alleles, and for all possible combinations of substitutions introduced on the base allele, we threaded the corresponding protein sequences on the template structure. We then performed energy optimization and assessed the stability of the resulting models by calculating the peptide:HLA interface energies using the *Rosetta* software (38) (**Appendix Scripts 1-5**) and (**Figures 2C, D**). This allowed us to evaluate the effect of specific residues on the overall stability for each chimeric molecule and, subsequently, narrow down the selection of groove residues to a minimal set of substitutions that would confer binding to the provided peptide. As expected, for all cases the chimeric models were more stable than models of the threaded base sequence on the groove template, but less stable than the corresponding native groove structures (**Supplementary Table 3**). For the top 2.5% structures with the lowest energies, we calculated enrichment scores for each polymorphic position, which represent the fraction of top chimeric HLAs carrying a specific substitution for a groove allele residue. More specifically, positions with an enrichment score of 1.0 indicate substitutions that are present in all structures, whereas substitutions with very low or 0 enrichment scores most likely affect the overall stability of the pHLA complex and thus are not favorable (**Figure 2D**). Additionally, mutations conferring different chemical properties at a certain position, such as a charged in the place of a neutral residue and *vice versa*, were always included in the minimal set whereas mutations replacing similar residues were excluded. To limit the number of substitutions impacting the TCR surface of the base allele, mutations in PTB positions were considered only if they contained a heavy atom within a more stringent threshold of 3.5 Å from the peptide. For the experimental validation of the designed chimeric HLAs, we performed previously established protein refolding (54) using groove-specific peptides, stability measurements by differential scanning fluorimetry (DSF) analysis (55), and peptide binding assays *in vitro* (56) (**Figure 2E**). Our proposed rational approach for exploring combinations of groove specificities and TCR contact surfaces from naturally occurring MHC-I alleles provides the means to study the principles of pMHC-I/TCR recognition and assess TCR cross-reactivity, with important biomedical ramifications in the design of peptide-centric therapeutics.

### Altering B- and F-Pocket specificities on HLA-A*02:01

**Figure 3:**
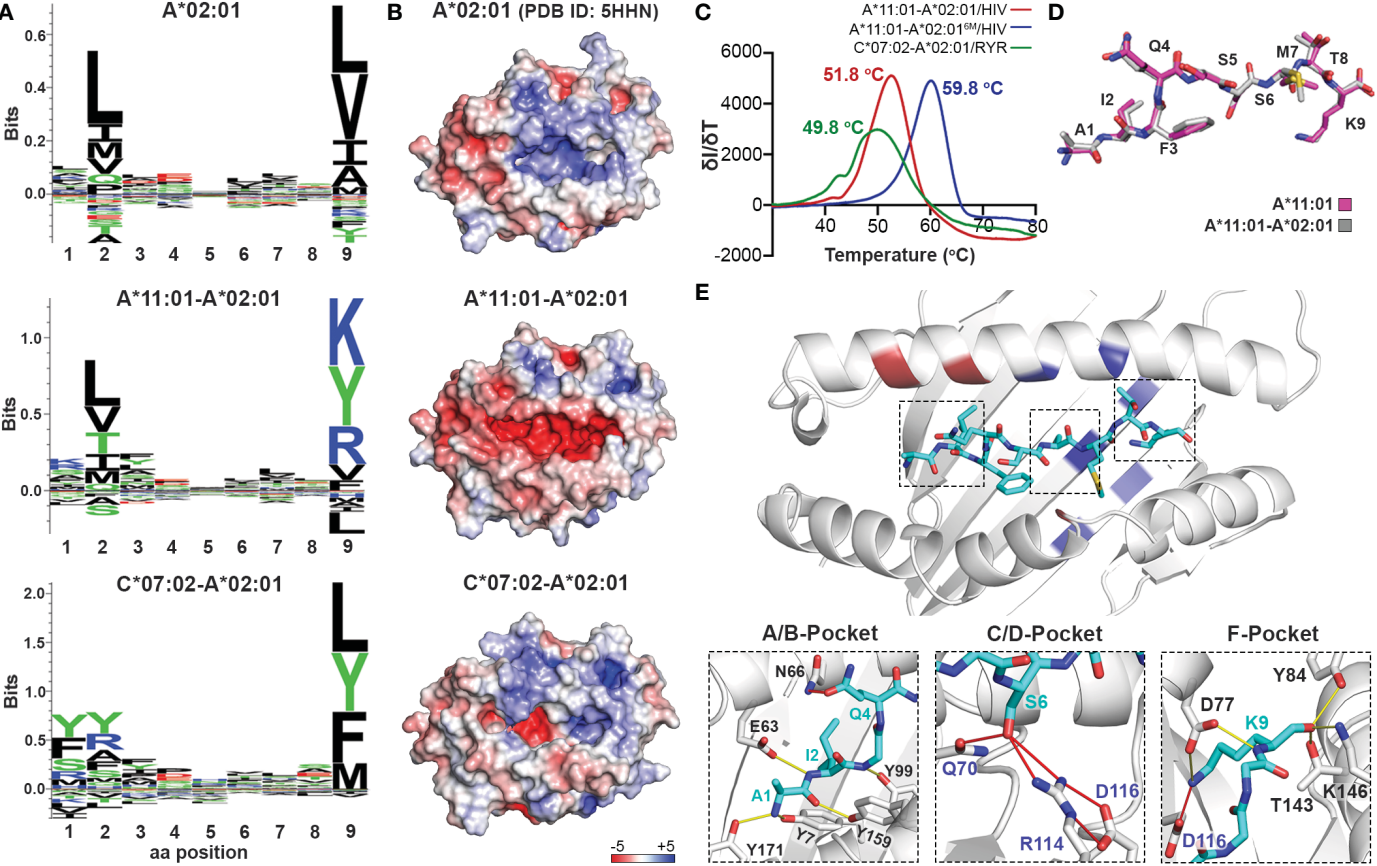
Production of chimeric HLA-A*02:01 peptide complexes with altered B- or F-pocket specificities according to A*11:01 or C*07:02 structural templates. **(A)** Sequence logos of HLA- A*02:01, A*11:01-A*02:01, and C*07:02-A*02:01 molecules rendered using an in-house protocol and visualized in Seq2Logo from the NetMHCpan4.0 (40). **(B)** Electrostatic surface potential analysis for HLA-A*02:01 (PDB ID: 5HHN), A*11:01-A*02:01, and C*07:02-A*02:01 calculated using the APBS solver in PyMOL. In all panels, the electrostatic surface potential is shown as a range between +5 kT/e (in blue) to -5 kT/e (in red) representing positive and negative charges, respectively. k_B,_ Boltzmann constant; T, temperature; e, unit charge. **(C)** Thermal stabilities of HLA-A*11:01-A*02:01/HIV-1 RT (red), A*11:01-A*02:01^6M^/HIV-1 RT (blue), and C*07:02-A*02:01/RYR (green). Data are mean ± SD obtained for n = 3 technical replicates. **(D)** Overlay of the HIV-1 RT peptide bound to the chimeric HLA-A*11:01-A*02:01 (grey) and wild-type A*11:01 (magenta) molecules. **(E)** Crystal structure of the HLA-A*11:01-A*02:01/HIV-1 RT complex. Substitutions of the HLA-A*11:01-A*02:01 (red and blue) and A*11:01-A*02:01^6M^ (blue) chimeras are highlighted. Hydrogen bonds and salt bridges between the peptide and the base or groove allele residues are represented by yellow or red lines, respectively. Peptide, A*02:01-specific, and A*11:01-specific residues are labeled in cyan, black, and blue font, respectively.

Considering that the primary anchor positions for peptide binding onto MHC-I molecules are the P2 and P9 (20), we employed the CHaMeleon approach to design synthetic pMHC-I molecules with altered peptide specificities by changing the B- and F-pockets of a base allele. For this purpose, we used the common human HLA-A*02:01 allotype as base with a preference for hydrophobic residues at positions P2 and P9 (**Figure 3A** and **Supplementary Figure 1A**). As structural templates, we used the previously defined X-ray structures of HLA-A*11:01 (PDB ID: 1Q94) and C*07:02 (PDB ID: 5VGE) together with the high affinity, immunodominant peptide antigens HIV-1 RT (AIFQSSMTK) and RYR (RYRPGTVAL), respectively. These alleles show distinct peptide specificities with a preference for the charged Lys/Arg residues in the P9 anchor for HLA-A*11:01, and aromatic or charged residues in the P2 anchor for C*07:02 (**Supplementary Figure 1B**). We identified and substituted 9 and 14 residues from HLA-A*11:01 and C*07:02 within the A*02:01 groove to generate the HLA-A*11:01-A*02:01 and C*07:02-A*02:01 chimeras, respectively (**Table 1**). We next predicted the peptide specificities of the chimeric molecules (see Methods) and confirmed that the introduced amino acid substitutions resulted in altered peptide-binding specificities in positions P2 and P9, to resemble the sequence of the groove alleles (**Figure 3A**). Comparison of the calculated energy values of the threaded structures showed that in both cases the chimeric molecules were more stable than the base but not the groove alleles (**Supplementary Table 3**). Electrostatic surface potential analysis using the *Rosetta* models of each designed chimeric MHC-I, revealed altered surface charges of the HLA-A*02:01 groove, which are known to play a crucial role in selective peptide binding (35). As expected, the groove of HLA-A*11:01-A*02:01 was negatively charged, while HLA-C*07:02-A*02:01 changed to negatively charged A- and B-pockets but maintained a positively charged F-pocket (**Figure 3B**).

To experimentally validate the designed chimeric HLAs, we refolded HLA-A*11:01-A*02:01 and C*07:02-A02:01 with the HLA-A*11:01-specific HIV-1 RT and HLA-C*07:02-specific RYR peptides, respectively. In both cases we were able to purify recombinant pMHC-I complexes by SEC (**Supplementary Figure 1C**) and further DSF analysis revealed melting temperatures characteristic of properly conformed peptide-bound molecules (T_m_=51.8°C for A*11:01-A*02:01/HIV-1 RT and 49.8°C for C*07:02-A*02:01/RYR, **Figure 3C**) (55). Taken together, our SEC and DSF results revealed that HLA groove-specific mutations can form properly folded and stable chimeric pMHC-I molecules after introducing target groove-specific peptides. We then sought to determine whether these peptides adopted a similar conformation compared to their parental template HLA, considering that the conformation and mobility of the bound peptide could affect the affinity for TCR recognition (32, 57). While we attempted to solve the crystal structures for both complexes, diffraction-quality crystals were obtained solely for the HLA-A*11:01-A*02:01/HIV-1 RT chimera. The best crystal diffracted to a 2.02 Å resolution and had clear electron density for the HIV-1 RT peptide, which we modeled in the F_0_-F_c_ electron density map (**Table 2** and **Supplementary Figure 2**). Overlay of the HIV-1 RT peptide from the wild-type HLA-A*11:01 versus the chimeric pMHC-I complex, revealed that both peptides adopted an identical backbone conformation with a deviation of 0.543 Å in RMSD values (**Figure 3D** and **Supplementary Table 4**). Additionally, we observed that while the B-pocket was occupied by Ile2 which was principally stabilized through hydrogen bonds with the peptide main chain, the F-pocket was occupied by Lys9 projecting directly into the HLA groove (**Figure 3E**). The observed accommodation of Lys9 into the F-pocket was the result of two salt bridge interactions between the Lys side chain and the introduced HLA-A*11:01 groove-specific residues Asp74 and Asp116 (**Figure 3E**). These residues appeared to orient and stabilize the Lys9 side chain within the groove, while the main chain was further stabilized by hydrogen bonds with the HLA-A*11:01-specific Asp74 and A*02:01-specific Tyr84, Thr143, Lys146, and Trp147 (**Figure 3E**). Interestingly, the introduced mutations Gln70 and Arg114 were responsible for forming multiple hydrogen bonds with Ser6 of the peptide within the C/D-pocket (**Figure 3E**). While we identified distinct HLA-A*11:01 groove-specific mutations crucial for peptide binding, several residues did not appear to be necessary for peptide association. We, thus, hypothesized we could optimize and refine the HLA-A*11:01-A*02:01 chimera, by re-engineering the HLA-A*02:01 base to introduce only six groove-specific mutations as opposed to the previous nine. This new six mutant HLA-A*11:01-A*02:01 (A*11:01-A*02:01^6M^) chimera was not only capable of refolding with the HIV-1 RT peptide (**Supplementary Figure 1C**) but was also significantly more stable (T_m_=59.8 °C) compared to the initial construct (T_m_=51.8 °C) (**Figure 3C**). Taken together, our HLA-A*11:01-A*02:01 structure revealed that the newly introduced peptide antigen adopted an identical conformation to that seen in the wild-type, parental HLA-A*11:01 structure (**Supplementary Table 4**) (58), further validating our fixed-backbone design approach. Finally, based on the observed interactions with the peptide backbone, our design could be further optimized to improve pMHC-I complex stability.

### Introducing a new P5 anchor within the C-Pocket of HLA-A*02:01

**Figure 4:**
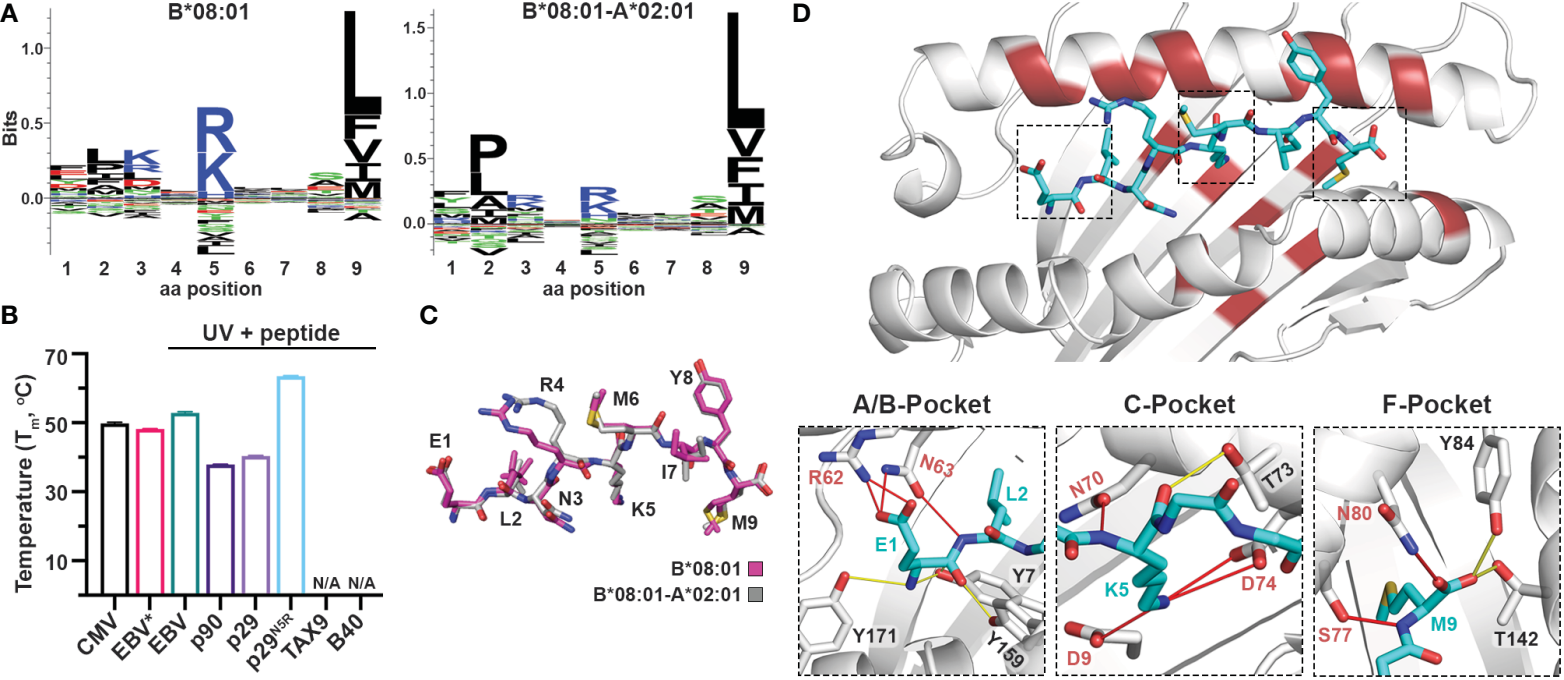
Introduction of a P5 anchoring specificity into the C-pocket of HLA-A*02:01 using a B*08:01 structural template. **(A)** The sequence logos of the HLA-B*08:01 (left), and B*08:01-A*02:01 (right) rendered using an in-house method and visualized in Seq2Logo from the NetMHCpan4.0 (40). **(B)** Thermal stabilities of HLA-B*08:01-A*02:01 refolded with CMV (ELNRKMIYM) or EBV* (FLRGRAJGL, where J is the 3-amino-3-(2-nitrophenyl)-propionic acid) and after UV-irradiation in the presence of 10-fold molar excess of EBV, p90, p29, p29^N5R^, TAX9, and B40 peptides. Data are mean ± SD obtained from n = 3 technical replicates. N/A, no exchange. **(C)** Overlay of the CMV peptide bound to the chimeric B*08:01-A*02:01 (grey) and wild-type B*08:01 (magenta) molecules. **(D)** Crystal structure of HLA-B*08:01-A*02:01/CMV complex where substitutions of the groove residues are highlighted in red. Hydrogen bonds and salt bridges between the peptide and the base or groove allele residues are shown as yellow or red lines, respectively. Peptide, A*02:01-specific, and B*08:01-specific residues are labeled in cyan, black, and red font, respectively.

Naturally occurring HLA molecules can bind and display a wide distribution of peptide sequences (termed peptide repertoires), that consist of polar, hydrophobic, or charged amino acids at defined anchor positions. However, the peptide pools presented by known alleles do not cover the entire range of amino acid combinations on a peptide sequence, implying that the displayed repertoire at the population level contains blind spots of ‘forbidden’ peptides (22). Thus, we explored further the applications of the CHaMeleon workflow to modify the set of binder peptides of an HLA molecule of interest, by introducing novel anchor positions within the HLA-A*02:01 groove. For this purpose, we selected HLA-B*08:01 with a distinct preference for peptides with charged residues (Arg/Lys) at position P5 (**Figure 4A**). To generate the HLA-B*08:01-A*02:01 chimera, a minimal set of 18 B*08:01-specific residues was identified and substituted within the A*02:01 groove based upon *Rosetta* threading and binding energy analysis, using the crystal structure of wild-type HLA-B*08:01 refolded with the CMV (ELNRKMIYM) peptide as a modeling template (PDB ID: 4QRT; **Table 1**). We experimentally validated the ability of the designed chimeric HLA to form stable protein complexes with the desired CMV peptide, using *in vitro* refolding, purification and DSF analysis which revealed a T_m_ of 49.8°C (**Supplementary Figure 1D** and **Figure 4B**).

We next examined whether the HLA-B*08:01-A*02:01 chimera could recapitulate the peptide-binding specificity of the groove allele we used as a structural template, namely HLA-B*08:01. We selected the HLA*B:08:01 specific CMV and EBV (FLRGRAYGL), the A*02:01 specific TAX9 (LLFGYPVYV) and p90 (RLRGVYAAL), and the B*40:01 specific B40 (TEADVQQWL) peptides, as well as the H2-L^d^ specific p29 (YPNVNIHNF) epitope from the HIV gp120 protein, based on established epitopic sequences that were further validated by NetMHCPan4.0 predicted binding affinities (**Supplementary Table 5**). We then refolded the chimeric HLA with a B*08:01-specific photolabile peptide (EBV* = FLRGRAJGL, where J is the 3-amino-3-(2-nitrophenyl)-propionic acid) (43) with a T_m_=48.2°C, to perform UV-mediated peptide exchange experiments (**Supplementary Figure 1D** and **Figure 4B**) (44). Incubation with 10-fold molar excess of peptide followed by UV-irradiation led to an up-shift in the T_m_ peak for EBV (T_m_=52.9°C) (**Figure 4B**), indicating the formation of stable pMHC-I molecules. Contrariwise, the p29 weak-binder peptide was unable to exchange (T_m_=40.4°C), demonstrating that the chimeric HLA groove is selective for HLA-B*08:01-specific peptides (**Figure 4B**). Based on the sequence logo for HLA-B*08:01 peptide specificity profile (**Figure 4A**), we hypothesized that introduction of a charged residue in P5 of the weak-binder p29 peptide would enhance binding, and therefore designed the mutant peptide N5R p29 (p29^N5R^, YPNVRIHNF). Notably, peptide exchange experiments with HLA-B*08:01-A*02:01/FLRGRAJGL and excess of the mutant peptide resulted in a thermal shift of 23°C compared to p29 (T_m_=63.6°C vs. 40.4°C, **Figure 4B**), suggesting the formation of stable complexes. The p90 peptide showed very little exchange with a T_m_ of 37.9°C, while the A*02:01- and B*40:01-specific peptides TAX9 and B40 were unable to exchange (**Supplementary Table 6**). Altogether, our peptide exchange data further support that the HLA-B*08:01-A*02:01 chimera can preferably bind epitopes with high affinity for the binding groove of the template allele, namely B*08:01.

While we were able to demonstrate that a synthetic MHC-I molecule with an additional P5 anchor could be designed and refolded, whether the B*08:01-specific peptide adopted an identical conformation compared to the wild-type template allele remained to be evaluated. Hence, we attempted to solve the structure of HLA-B*08:01-A*02:01/CMV complex in an I23 space group and obtainedcrystals which diffracted to a 2.72 Å resolution (**Table 2**). As in the HLA-A*11:01-A*02:01 crystal structure, we observed unambiguous electron densities for the CMV peptide that we modeled within the F_0_-F_C_ electron density map (**Supplementary Figure 3**). Overlay of the CMV peptide bound to the wild-type HLA-B*08:01 and the B*08:01-A*02:01 chimera revealed an identical backbone conformation with a deviation of 0.495 Å in RMSD values between the two structures (**Figure 4C** and **Supplementary Table 4**), in agreement with our previous results for the HLA-A*11:01-A*02:01 chimera. While the F-pocket was occupied by Met9 and stabilized by hydrogen bonds along the main chain, the A-pocket was occupied by Glu1 which side chain interacted with the B*08:01-specific residues Arg62 and Asn63 (**Figure 4D**). A strong electron density was observed for Lys5 within the C-pocket which formed three salt bridge interactions and one hydrogen bond with the B*08:01-specific residues Asp9, Asn70 and Asp74 (**Figure 4D**), suggesting that these residues are crucial for stabilizing the peptide within the HLA groove. Altogether, these findings support the introduction of a novel P5 anchor within the HLA-A*02:01 groove to generate a chimeric molecule with a distinct peptide repertoire, without affecting the adopted conformation of the bound peptide.

### Use of chimeric HLAs as molecular probes for identifying peptide-centric receptors

**Figure 5:**
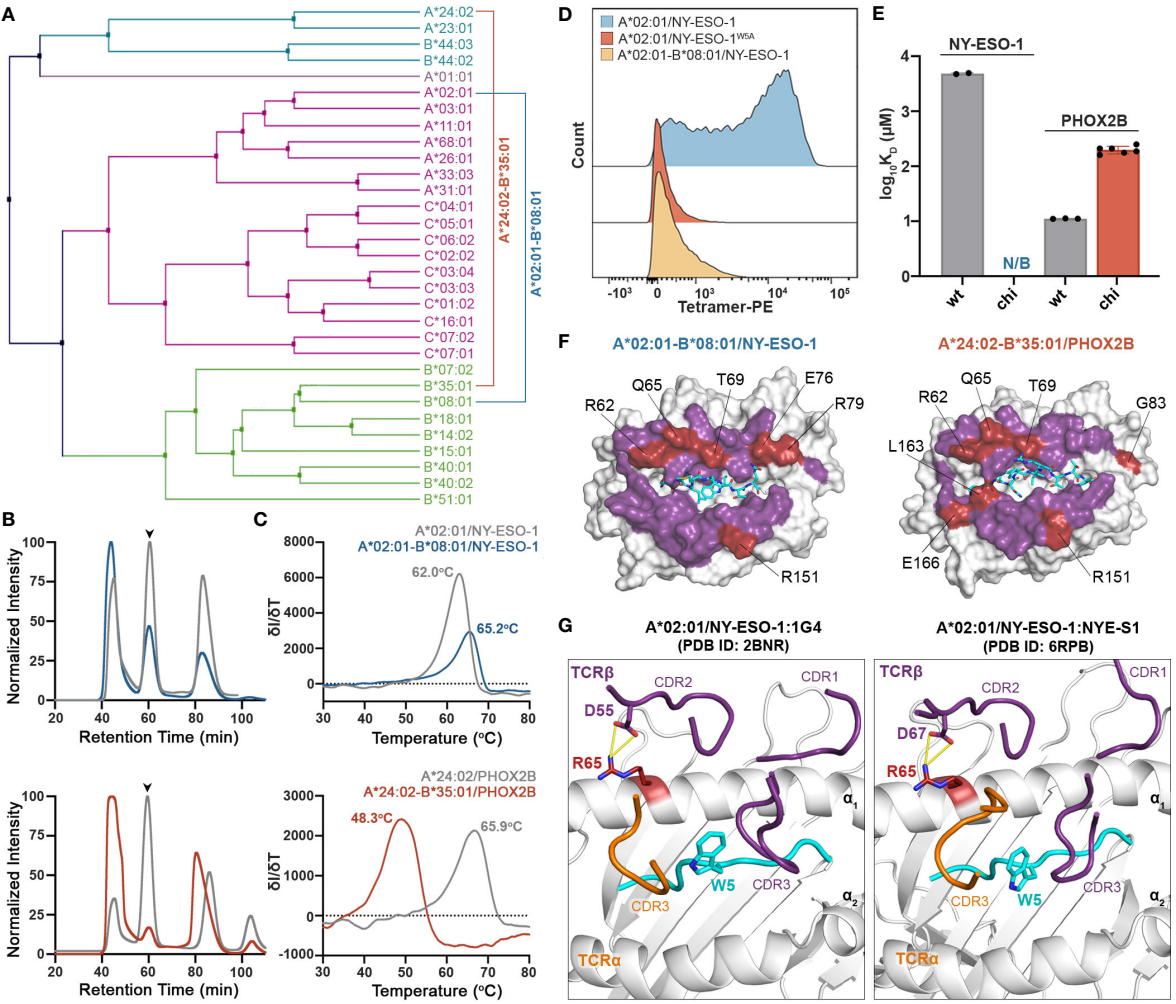
Application of chimeric HLAs as probes for assessing peptide-centric interactions with immune receptors for targeted therapy. **(A)** Phylogenetic analysis of a divergent set of common HLA allotypes using the TCR contacting residues to define sequence similarity. **(B)** SEC traces of recombinant HLA-A*02:01/NY-ESO-1, A*02:01-B*08:01/NY-ESO-1, A*24:02/PHOX2B, and A*24:02-B*35:01/PHOX2B molecules. The protein peaks are indicated by the arrows. **(C)** Melting temperatures (T_m_, °C) of the pMHC alleles in **(B)** determined by DSF experiments. Data are mean ± SD obtained for n = 3 technical replicates. **(D)** Staining of 1G4-transduced primary CD8+ T cells with PE-conjugated tetramers of A*02:01 presenting the wild-type NY-ESO-1 or the mutated NY-ESO-1^W5A^ peptides, and the chimeric A*02:01-B*08:01/NY-ESO-1 complex. Staining was observed only in the case of A*02:01/NY-ESO-1, suggesting positive recognition by the TCR, whereas in the case of the negative control and the chimeric pMHC the interactions are disrupted. **(E)** Comparison of the SPR determined K_D_ values for NYE-S1 and 10LH interacting with HLA-A*02:01 and A*24:02 wild-type and chimeric molecules presenting NY-ESO-1 and PHOX2B peptides, respectively. Data are mean ± SD for n = 2 (NYE-S1) or n = 3 (10LH) technical replicates. K_D_, equilibrium constant; N/B, no binding. **(F)** Surface structure of the *Rosetta* model of HLA-A*02:01-B*08:01/NY-ESO-1 and A*24:02-B*35:03/PHOX2B chimeras, where all TCR-contact residues are highlighted (purple and red). The wild-type B*08:01 or B*35:01 residues are shown in red. **(G)** Structural comparison of the HLA-A*02:01/NY-ESO-1 complex bound by the TCRs 1G4 (PDB ID: 2BNR) and NYE-S1 (PDB ID: 6RPB). The HLA-A*02:01, the NY-ESO-1 peptide, and the TCR-α and -β chains are colored in white, cyan, orange, and purple, respectively. The identified Arg65 is represented as a single stick (in red) and its interactions with TCR-β chain are in yellow.

We next sought to address whether we can use chimeric HLAs to evaluate the extent to which interactions with specific TCRs or therapeutic antibodies are dependent upon interactions with HLA framework residues. Towards this goal, we tested the wild-type TCRs 1G4 (31) and NYE-S1 (30) which recognize the tumor epitope NY-ESO-1 (SLLMWITQV) on HLA-A*02:01, as well as the peptide-centric engineered CAR 10LH that targets the neuroblastoma peptide PHOX2B (QYNPIRTTF) presented by A*24:02 (34). To design chimeric HLAs able to bind these epitopes on their non-physiological base we, first, performed a phylogenetic analysis of the TCR contacting residues of selected *HLA-A*, *-B*, and *-C* allotypes to identify alleles with the most dissimilar TCR interacting surfaces compared to HLA-A*02:01 and A*24:02 (**Figure 5A**). Based on our analysis, we selected HLA-B*08:01 and B*35:01 to generate the HLA-A*02:01-B*08:01 and HLA-A*24:02-B*35:01 chimeras presenting the NY-ESO-1 and PHOX2B peptide antigens, respectively. Using the CHaMeleon approach, we identified and introduced 11 HLA-A*02:01 and 16 A*24:02 residues in the peptide-binding grooves of B*08:01 and B*35:01, respectively (**Table 1**). Both chimeric molecules were successfully refolded with their respective target peptides (**Figure 5B**) and, notably, the HLA-A*02:01-B*08:01 chimera was able to form a more stable complex with NY-ESO-1 compared to the wild-type A*02:01 (T_m_=65.2°C vs. T_m_=62.0°C), as revealed by DSF experiments (**Figure 5C**). Contrariwise, the HLA-A*24:02-B*35:01 chimera was destabilized by almost 15 °C compared to the wild-type A*24:02 (T_m_=48.3°C vs. T_m_=65.9°C), although was still able to form loaded pMHC-I complexes (**Figure 5C**).

To test our hypothesis, we stained primary CD8+ T cells transduced with the wild-type TCR 1G4 that recognizes the NY-ESO-1 peptide presented by A*02:01 (31) (**Supplementary Figure 4**), and generated phycoerythrin (PE) tetramers of HLA-A*02:01/NY-ESO-1 and A*02:01-B*08:01/NY-ESO-1, as previously described (59). As a negative control, we used HLA-A*02:01 refolded with the NY-ESO-1 peptide carrying an Ala substitution in position 5, namely NY-ESO-1^W5A^ (SLLMAITQV), which has been shown to be essential for TCR recognition (60). Analysis by flow cytometry revealed lack of staining with HLA-A*02:01/NY-ESO-1^W5A^ and A*02:01-B*08:01/NY-ESO-1 compared to the wild-type A*02:01/NY-ESO-1 tetramers (**Figure 5D**). These results confirm that TCR 1G4 recognizes specific peptide:HLA antigens in a highly restricted manner (61), as interactions were disrupted both in the case of the wild-type MHC-I presenting a peptide with a single amino acid substitution and the chimeric pMHC-I presenting the target peptide. We, next, used the newly characterized NYE-S1 TCR selective for HLA-A*02:01/NY-ESO-1 (30) to quantitively assess pMHC-I/TCR interactions using surface plasmon resonance (SPR) experiments. Soluble NYE-S1 bound weakly to immobilized HLA-A*02:01/NY-ESO-1 with a dissociation equilibrium constant K_D_ = 4.9 μM, in agreement with previous studies (30), but was unable to interact with both HLA-A*02:01/NY-ESO-1^W5A^ and A*02:01-B*08:01/NY-ESO-1 chimeric molecules (**Figure 5E** and **Supplementary Figures 5A, B**). Additionally, we tested the scFv-based CAR 10LH, which is selective for A*24:02/PHOX2B and has been shown to interact with this specific epitope even when presented by different HLAs, i.e. HLA-A*23:01 and B*14:02 (34). As a negative control, we used HLA-A*24:02 refolded with PHOX2B peptide carrying an Ala substitution in P6, namely PHOX2B^R6A^, which completely disrupts interactions with 10LH (34). As expected, 10LH bound to HLA-A*24:02 presenting the wild-type PHOX2B peptide with a K_D_ of 11.1 nM but not the mutated PHOX2B^R6A^ (**Figure 5E** and **Supplementary Figures 5C, D**). Notably, the chimeric HLA-A*24:02-B*35:01/PHOX2B and 10LH interactions were 20-fold weaker with an estimated nanomolar range K_D_ compared to the wild-type (**Figure 5E** and **Supplementary Figures 5E, F**). However, the observed 200 nanomolar binding still falls within the affinity range (up to micromolar) for TCRs/CARs and their pHLA targets which has been demonstrated to sufficiently trigger T cell killing (62, 63).

To explore the structural basis of the loss of TCR recognition for the chimeric pMHC-I molecules, we compared the TCR-interacting surfaces of the generated chimeric models. We observed that 6 out of 8 polymorphic TCR residues for HLA-A*02:01-B*08:01 and 7 out of 10 for A*24:02-B*35:01 chimeras were residues of the base allele and could, thus, affect TCR/CAR recognition (**Figure 5F**). To further determine which HLA-B*08:01 base residues were responsible for the loss of NYE-S1 recognition, we compared them to the A*02:01 residues responsible for TCR binding based on the solved crystal structures of HLA-A*02:01/NY-ESO-1 with the TCRs 1G4 and NYE-S1 (30, 31). We identified the HLA-A*02:01 residue Arg65 to be important for 1G4 and NYE-S1 binding along the α_1_ helix, forming interactions with Asp55 and Asp67 of the CDR2β loops, respectively (**Figure 5G**). In HLA-A*02:01-B*08:01 chimera, this residue was replaced by Gln65 of the wild-type B*08:01, suggesting that disruption of these interactions is crucial for TCR binding. Interestingly, the same position differs between HLA-A*24:02 and B*35:01 (**Figure 5F**), however had no effect on 10LH recognition, as expected for the peptide-centric CARs which are not constrained by the germline-encoded CDR1-2/MHC interactions. Taken together, our cell-based and biophysical data confirm that the peptide antigen alone is not sufficient to maintain known pMHC-I/TCR interactions when presented in the context of a divergent HLA framework surface and suggest that loss of binding can occur even with a single amino acid substitution on the MHC-I/TCR interacting surface. In contrast, recognition by the peptide-centric CAR 10LH was not disrupted, highlighting the potential of scFV-based immunotherapies to target a broad range of allotypes.

## Discussion

The highly polymorphic nature of the MHC-I peptide binding groove highlights a stable structural scaffold which can be adapted to accommodate a diverse panel of ligands (6). While human MHC-I allotypes encompass a plethora of peptide binding specificities, there remain gaps in the repertoire of antigens which can be recognized and displayed by the existing HLA proteins (20, 22). On the other hand, TCRs can recognize different peptide:MHC-I complexes through a combination of peptide-centric and germline contacts with MHC-I framework residues and are limited to a restricted range of interactions with HLAs. Here, we outline a systematic approach to generate synthetic MHC-I molecules blending desired peptide and TCR interaction properties. Our analysis shows that we can use existing structural information to discern MHC-I residues responsible for peptide binding and TCR recognition, enabling us to design chimeric molecules according to a fixed-backbone protocol that is guided by a structural template. We provide biochemical evidence that the HLA pockets within the groove can be altered to accommodate new peptides while maintaining the TCR surface features of a specific HLA allotype. Our approach is further validated by the solved crystal structures for two chimeric MHC-I molecules, which reveal that the peptide is presented in the specified conformation. Notably, all-atom RMSD values between the crystal structure and the *Rosetta* model were below 2 Å both for the peptide and MHC-I α_1_/α_2_ domains (**Supplementary Table 4**). Finally, functional characterization using *in vitro* tetramer staining and biophysical binding experiments demonstrates the practical utility of our chimeric molecules as screening tools to evaluate peptide-centric interactions with T cell receptors and therapeutic antibodies, respectively.

Our work offers insights into principles underpinning the molecular evolution of MHC-I allotypes, and the emergence of distinct supertypes (7). Owning to the stability and malleability of the MHC-I scaffold, a minimal set of amino acid substitutions can lead to drastic changes in peptide binding preference, and thereby supertype divergence (64). It is worth noting that for some of the chimeric molecules designed in our study, we can identify known HLA allotypes with similar peptide-binding groove sequences and assumed peptide binding preferences. In particular, the HLA-A*11:01-A*02:01 chimera, designed to accommodate peptides with positively charged P9 residues, is similar in sequence (4 amino acid differences among peptide-binding residues) to the known allotypes HLA-A*03:05 and A*03:17 (A03 supertype) (64) that have acidic F-pockets, and therefore are predicted to bind positively charged peptides (**Supplementary Figure 6**). Likewise, the designed HLA-A*11:01-A*02:01^6M^ chimera possessing the groove of A*11:01 (A03 supertype), differs in 4 peptide-binding residues with each of the HLA-A*02:35 and A*02:246 allotypes (A02 supertype) (64) (**Supplementary Figure 6**). Interestingly, a combination of all substitutions from the wild-type alleles, where two of them are shared, results in our computationally designed chimeric sequence (**Supplementary Figure 6**). This in turn suggests that our synthetic molecules incorporate features from distinct supertypes that could naturally occur over time and represents an example of convergent evolution between A03 and A02 supertypes. However, there is no structural evidence that these allotypes bind the peptides in a similar backbone conformation compared to the wild-type template allele. Our study also describes a chimeric HLA, namely HLA-B*08:01-A*02:01, with no direct equivalent amongst naturally occurring HLAs (15 amino acid differences with the closest allotype). This could be either due to lack of sequence data on already existing allotypes in the population, or because this specific peptide binding motif has not yet been sampled by the ongoing evolutionary process for A02 alleles. In summary our designed molecules provide evidence that barriers between different supertypes are low and provide an avenue for creating novel allotypes which are not represented in the existing HLA repertoires.

Chimeric MHC molecules designed with desired peptide-binding grooves and TCR-interacting surfaces have potential immune system engineering applications towards the development of targeted therapies for breaking tolerance for weak disease- or cancer-associated antigens. Current approaches to break self-tolerance include the use of altered peptide ligands for personalized cancer vaccines (65, 66), and the introduction of checkpoint inhibitors to overcome peripheral tolerance (67). A recent study has shown that introduction of point mutations at the TCR binding interface of native MHCs presenting tumor-associated antigens can be used to activate T cells through allorecognition (33). Using the CHaMeleon approach outlined in this work, we can introduce novel anchor positions to the peptide-binding groove of selected MHCs and generate chimeric molecules presenting established tumor-associated antigens with modified TCR interaction surfaces, relative to a specific HLA allotype. These chimeric HLAs can be then used as immunogens, to elicit alloreactive T cell responses for self-antigens that are upregulated in cancer (68). In a similar manner, epitope-focused vaccination strategies are based on eliciting antibodies towards non-immunogenic antigens with multiple applications against diseases and cancer therapy (69, 70). More importantly, with the advent of CAR-T cell therapies (71), there has been an increasing interest in designing peptide-centric receptors that are highly specific for a certain peptide sequence and are relatively tolerant to amino acid substitutions of HLA framework residues within the peptide:MHC complex (34). As implied by our proof of concept *in vitro* binding studies, chimeric MHC-I molecules can serve as screening tools to identify peptide-centric CARs for specific antigens. When prepared in tetramerized form and used as selection markers in existing directed evolution and antibody panning approaches (72), chimeric peptide:MHC complexes can enable the development of therapies which can cover larger cohorts of patients.

Collectively, our results suggest that we are capable of re-capitulating and potentially expanding the antigen presentation profile of target alleles through a structure-guided, systematic redesign of the MHC-I peptide binding groove. Our approach serves as a toehold for understanding the molecular evolution and functional divergence of HLA allotypes, while also providing useful screening tools to facilitate the development of tolerance-breaking vaccines and targeted CAR-T therapies.

## Data availability statement

The datasets presented in this study can be found in online repositories. The names of the repository/repositories and accession number(s) can be found below: http://www.wwpdb.org/, 8ERX; http://www.wwpdb.org/, 8ESH.

## Author contributions

OA and NS conceived and designed the CHaMeleon workflow. OA performed phylogenic analysis of HLA sequences and analysis of existing database structures. TF, GP, and MY prepared and purified all recombinant MHC-I and TCRs. TF, GP, and MY performed DSF analysis of MHC-I complexes. TF and JD performed UV-irradiation photo-exchange and DSF analysis of MHC-I complexes. TF performed X-ray crystallography and structural analysis. TF and YS performed SPR analysis. JD and DD performed MHC-I tetramerization and flow cytometry analysis. NS acquired funding and supervised the project. GP, TF, and NS wrote the manuscript, with feedback from all the authors. All authors contributed to the article and approved the submitted version.
