# Supplementary material for: Decoupling peptide binding from T cell receptor recognition with engineered chimeric MHC-I molecules
Source: Front Immunol. 2023 Jan 25;14:1116906. doi: 10.3389/fimmu.2023.1116906 (PMC9905809; doi:10.3389/fimmu.2023.1116906)
Supplement: Supplementary file 1 [file DataSheet_1.pdf]

Supplementary Material

Decoupling peptide binding from T cell receptor recognition with engineered chimeric MHC-I molecules

Georgia F. Papadaki[1,2†], Omar Ani[1,†], Tyler J. Florio[1,2†], Michael C. Young[1,2], Julia N. Danon[1,2], Yi Sun[1,3], Devin Dersh[3], and Nikolaos G. Sgourakis[1,2*]

***Correspondence:**
Nikolaos G. Sgourakis
Nikolaos.Sgourakis@Pennmedicine.upenn.edu

This PDF file includes:

Supplementary Figures 1 to 6

Supplementary Tables 1 to 6

Appendices I-II

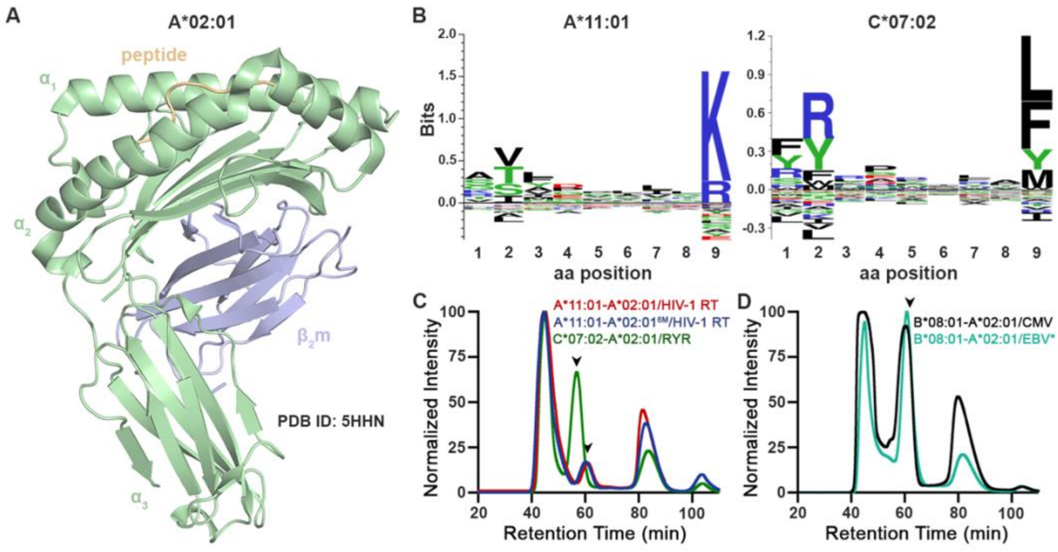

Supplementary Figure 1. Design and experimental validation of chimeric HLA-A*02:01 peptide complexes. (A) Crystal structure of HLA-A*02:01 where the α_{1-3} domains of the heavy chain (green), the light chain β_2m (purple) and the peptide (yellow) are depicted (PDB ID: 5HHN). **(B)** Sequence logo of peptides specific for HLA-A*11:01 (left), and HLA-C*07:02 (right), rendered using Seq2Logo in NetMHCpan4.0 (1). SEC traces of **(C)** HLA-A*11:01-A*02:01, HLA-A*11:01-A*02:01^{6M} refolded with the HIV-1 RT (AIFQSSMTK) peptide, C*07:02-A*02:01 refolded with the RYR (RYRPGTVAL) peptide, and **(D)** HLA-B*08:01-A*02:01 chimera refolded with the CMV (ELNRKMIYM) and EBV* (FLRGRAJGL) peptides. The protein peaks are indicated by the arrows. J, 3-amino-3-(2-nitrophenyl)-propionic acid.

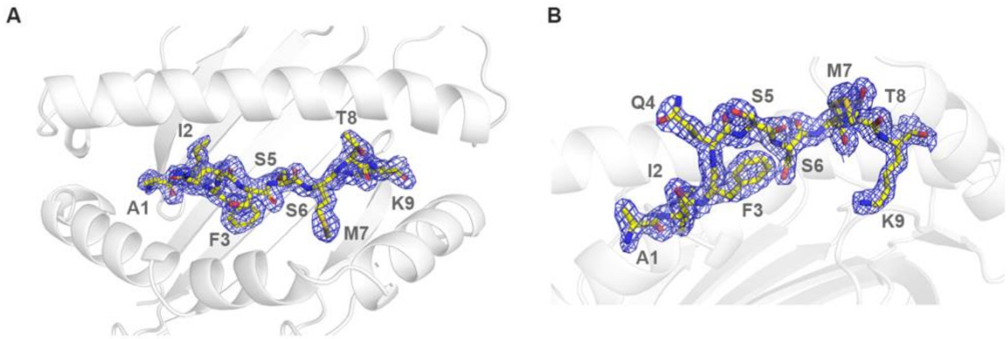

Supplementary Figure 2. Structure of the HIV-1 RT peptide presented by the HLA-A*11:01-A*02:01 chimera. (A and B) HLA-A*11:01-A*02:01 2Fo-Fc omit maps (blue) around the HIV-1 RT peptide (AIFQSSMTK; yellow) contoured at 1.3 sigma.

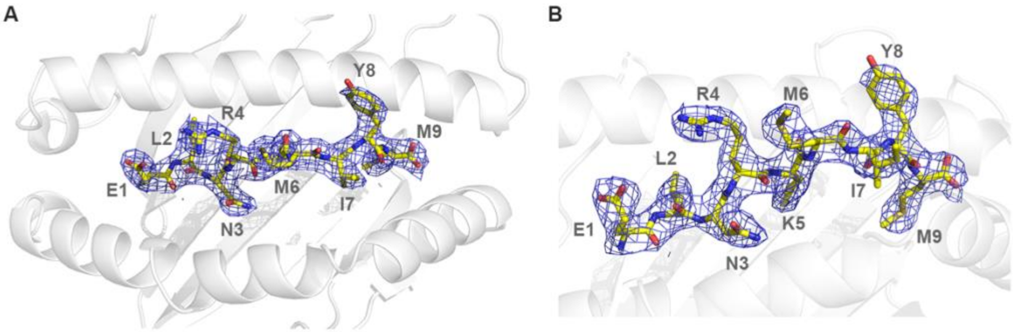

Supplementary Figure 3. Structure of the CMV peptide presented by the HLA-B*08:01-A*02:01 chimera. (A and B) HLA-B*08:01-A*02:01 2Fo-Fc omit maps (blue) around the CMV peptide (ELNRKMIYM; yellow) contoured at 1.0 sigma.

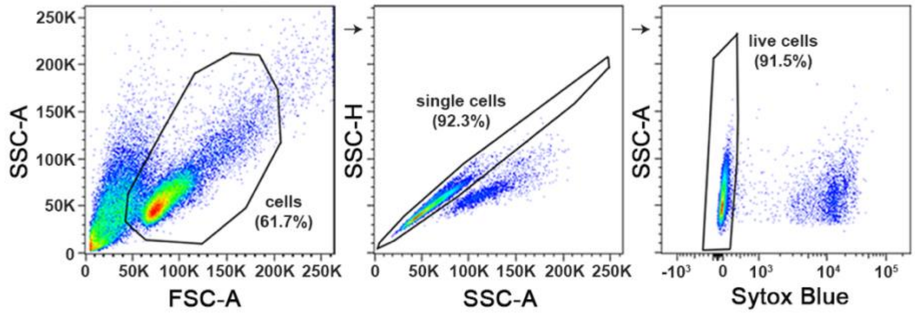

Supplementary Figure 4. Flow cytometry gating strategy of CD8+ T cells transduced with 1G4. Previously transduced or non-transduced primary human CD8+ T cells were thawed and recovered prior to tetramer staining. Cells were first sorted by side and forward scatter (SSC-A and FSC-A) followed by single cell isolation (SSC-A versus SSC-H plot). Gating for live cells was determined by Sytox blue staining and transduction efficiency was determined by staining with an anti-Vβ13.1-APC antibody (Miltenyi Biotec). Gates are shown in black and the percentages of events that are gated in parentheses. Acquisition was performed on LSR Fortessa (BD), and the data analyzed by FlowJo v10.8.1.

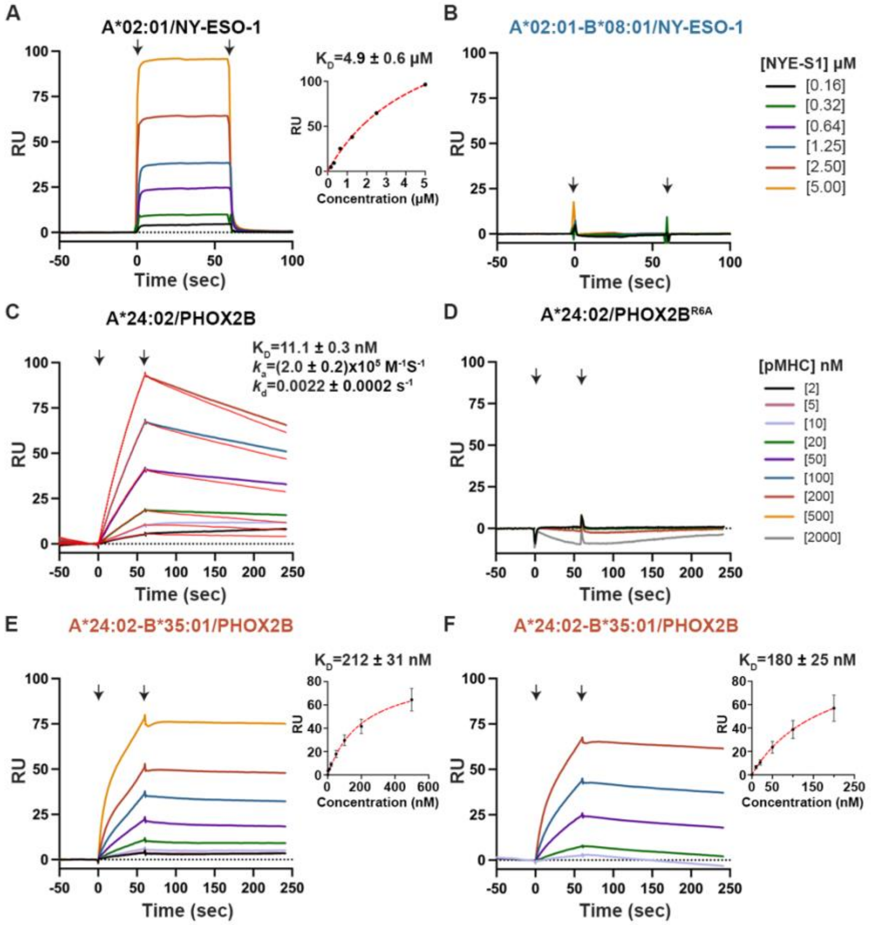

Supplementary Figure 5. TCR recognition dependence on interactions with MHC-I framework residues compared to peptide-centric CARs. SPR sensorgrams of vary concentrations of soluble NYE-S1 receptor flowed over a streptavidin chip coupled with **(A)** A*02:01/NY-ESO-1 and **(B)** A*02:01-B*08:01/NY-ESO-1. SPR sensorgrams of various concentrations of **(C)** A*24:02/PHOX2B or **(D)** A*24:02/PHOX2B^{R6A} as negative control flowed over a streptavidin chip coupled with the biotinylated scFv 10LH. Fits from the kinetic analysis are shown with red lines. **(E)** and **(F)** Two independent replicates of chimeric A*24:02-B*35:01/PHOX2B complex flown over a streptavidin chip conjugated with biotinylated 10LH. The K_D is an estimation since binding saturation is not reached. Data are mean ± SD for n = 2 (A, B), n = 3 (C-F) technical replicates. Injection and washing start points are indicated by arrows. K_D, equilibrium dissociation constant; k_a, association rate constant; k_d, dissociation rate constant; RU, resonance units.

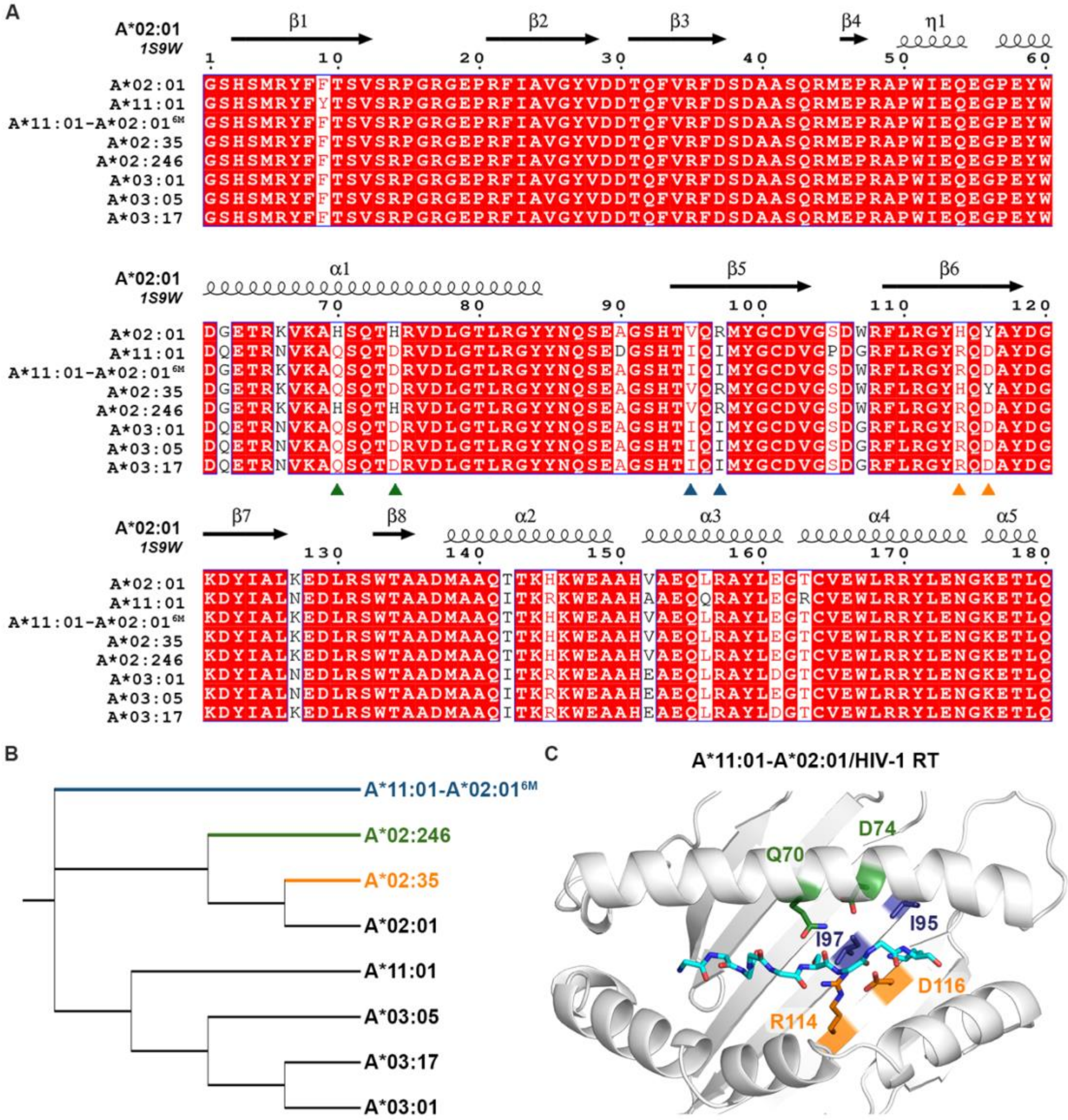

Supplementary Figure 6. Convergent evolution of A02 and A03 supertypes. (A) Sequence alignment of the α_1 and α_2 helices (residues 1-180) of A*11:01-A*02:01^{6M} with the template A*11:01, the base A*02:01, and the most similar to the chimeric molecules, known HLA allotypes. Substituted residues of the A*11:01-A*02:01^{6M} chimera that are different in A*02:246, A*02:35, or both are shown in green, orange, or blue arrowheads, respectively. Alignments were performed in ClustalOmega (2) and processed in ESPript (3). The structure of HLA-A*02:01 (PDB ID: 1S9W) was used as reference. Conserved residues are in red boxes. **(B)** Phylogenetic tree based on the sequence alignment in (A), using the Maximum Likelihood method in MEGA7 (4) and processed in iTOL (5). **(C)** The crystal structure of HLA-A*11:01-A*02:01/HIV-1 RT chimera where the indicated residues in (A) are highlighted, respectively.

Supplementary Table 1. Summary of the peptides used in this study.

Peptide Name	Allotype	Sequence
**TAX9**	A*02:01	LLFGYPVYV
**HIV-1 RT**	A*11:01	AIFQSSMTK
**NY-ESO-1**	A*02:01	SLLMWITQV
**NY-ESO-1^{W5A}**	A*02:01	SLLMAITQV
**p90**	A*02:01	RLRGVYAAL
**PHOX2B**	A*24:02	QYNPIRTTF
**PHOX2B^{R6A}**	A*24:02	QYNPIATTF
**CMV**	B*08:01	ELNRKMIYM
**EBV**	B*08:01	FLRGRAYGL
**EBV***	B*08:01	FLRGRAJGL
**p29^{N5R}**	B*08:01	YPNVRIHNF
**B40**	B*40:01	TEADVQQWL
**RYR**	C*07:02	RYRPGTVAL
**p29**	H2-Ld	YPNVNIHNF

Supplementary Table 2. Summary of the peptide-contact and TCR-contact frequencies (%) for each position P, as calculated from analysis of 384 pMHC-I structures from the Protein Data Bank (HLA3DB; https://hla3db.research.chop.edu/) and 36 pMHC-TCR structures from the ATLAS database (6). Peptide-only binding (PB) positions have a non-zero peptide-contact frequency and a TCR-contact frequency less than 10%, TCR-only binding (TB) positions have a non-zero TCR-contact frequency and a peptide-contact frequency less than 10%, and peptide-TCR-binding (PTB) have both frequencies greater than 10%. The consensus score for each category was calculated from the analysis of 2896 sequences HLAs curated from the IMGT/HLA database (7).

**Peptide-only binding (PB) positions**			
**Residue Position**	**Peptide-contact Frequency (%)**	**TCR-contact Frequency (%)**	**Consensus Score (%)**
**5**	13.90	0	99.79
**7**	94.01	0	99.76
**9**	39.65	0	53.79
**22**	0.78	0	99.90
**24**	11.81	0	63.98
**34**	0.19	0	99.69
**36**	0.36	0	99.69
**45**	39.28	0	38.20
**59**	19.55	8.11	99.79
**63**	94.11	2.70	68.82
**67**	32.65	0	29.52
**74**	13.54	0	57.41
**77**	95.83	0	46.66
**80**	73.89	5.41	45.01
**81**	17.31	0	79.99
**84**	78.24	0	99.55
**95**	11.10	0	48.84
**97**	34.64	0	53.09
**99**	80.71	0	85.93
**114**	16.19	0	38.96
**116**	36.55	0	35.15
**117**	0.83	0	99.83
**118**	0.02	0	99.83
**123**	18.63	0	99.86
**124**	0.26	0	99.79
**133**	1.70	0	99.93

**139**	0.05	0	99.45
**142**	0.07	0	89.84
**143**	44.39	0	96.20
**156**	42.42	0	57.10
**171**	94.53	0	93.05
**TCR-only binding (TB) positions**			
**Residue Position**	**Peptide-contact Frequency**	**TCR-contact frequency**	**Consensus Score**
**55**	0	2.70	99.72
**58**	0.21	21.62	99.55
**61**	0	2.70	99.83
**65**	6.08	86.49	67.30
**68**	0	45.95	99.69
**72**	0.92	86.49	99.76
**75**	0	32.43	99.59
**79**	0	16.22	75.87
**83**	0	8.11	77.15
**108**	0	2.70	99.59
**149**	0	21.62	96.58
**150**	6.46	83.78	97.86
**151**	0	67.57	75.32
**154**	0	70.27	99.83
**157**	0	10.81	99.65
**158**	0	70.27	94.64
**161**	0	10.81	97.03
**162**	0	18.92	99.59
**166**	0	40.54	92.46
**170**	6.01	16.22	99.59
**Peptide-TCR binding (PTB) positions**			
**Residue Position**	**Peptide-contact Frequency**	**TCR-contact frequency**	**Consensus Score**
**62**	24.76	56.76	72.90
**66**	74.83	81.08	45.32
**69**	13.28	89.19	41.79
**70**	62.12	10.81	37.95
**73**	58.57	59.46	86.69
**76**	30.56	59.46	54.37
**146**	90.36	40.54	99.86
**147**	91.03	18.92	91.46

**152**	36.03	35.14	50.47
**155**	48.89	94.59	99.79
**159**	99.27	48.65	99.83
**163**	25.97	62.16	51.33
**167**	55.66	35.14	87.94

Supplementary Table 3. Total binding energy calculations for the base, template, and chimeric alleles threaded and relaxed through the template allele. The mean energy values from three independent models and the standard deviation are included. Structures were calculated using the *Rosetta* software (8).

Chimeric HLA	Groove allele	Chimeric allele	Base allele
**A*11:01-A*02:01**	-570.79 ± 5.65	-564.34 ± 4.82	-516.84 ± 7.33
**A*11:01-A*02:01^{6M}**	-566.36 ± 7.61	-566.54 ± 4.77	-517.86 ± 2.70
**B*08:01-A*02:01**	-604.76 ± 4.39	-582.95 ± 4.45	-528.06 ± 1.61
**C*07:02-A*02:01**	-554.74 ± 2.43	-533.65 ± 4.26	-525.71 ± 2.15
**A*24:02-B*35:01**	-588.38 ± 0.64	-592.66 ± 1.68	-473.09 ± 3.29
**A*02:01-B*08:01**	-597.52 ± 3.30	-499.83 ± 3.96	-495.08 ± 1.67

Supplementary Table 4. RMSD (Å) values for the solved crystal structures *vs*. the template allele and *vs*. the *Rosetta* model. The calculations were performed using the 'Super' command in PyMOL, with the appropriate residue selectors for the backbone and full atom and 0 refinement cycles.

	**Chimeric HLA crystal structure *vs.* Template HLA**					
	**pMHC-I**		**Peptide**		**MHC-I**	
**Chimeric HLA**	*Backbone*	*Full atom*	*Backbone*	*Full atom*	*Backbone*	*Full atom*
**A*11:01-A*02:01**	0.833	1.466	0.543	1.278	0.839	1.471
**B*08:01-A*02:01**	0.982	1.842	0.495	0.973	0.997	1.879

	**Chimeric HLA crystal structure *vs.* Rosetta generated chimeric HLA**					
	**pMHC-I**		**Peptide**		**MHC-I**	
**Chimeric HLA**	*Backbone*	*Full atom*	*Backbone*	*Full atom*	*Backbone*	*Full atom*
**A*11:01-A*02:01**	0.893	1.51	0.543	1.278	0.904	1.517
**B*08:01-A*02:01**	0.982	1.956	0.495	0.973	0.997	1.996

Supplementary Table 5. Summary of the peptides used for the HLA-B*08:01-A*02:01 UV-mediated exchange experiments. Binding affinities (nM) to the template, base, and chimeric molecules were calculated using NetMHCpan4.0 (9).

		HLA Binding Affinities (nM)			
**Peptide**	**Sequence**	**A*02:01**	**B*08:01**	**B*08:01-A*02:01**	**A*02:01-B*08:01**
**CMV**	ELNRKMIYM	5821.13	387.4	510.04	11431.2
**EBV**	FLRGRAYGL	184.04	7.03	49.16	2060.1
**p29**	YPNVNIHNF	25098.64	1321.77	206.65	16876.15
**p29^{N5R}**	YPNVRIHNF	29131.61	53.69	36.83	21036.91
**p90**	RLRGVYAAL	317.8	222.23	543.51	1197.41
**TAX9**	LLFGYPVYV	2.04	5262.3	5456.42	58.98
**B40**	TEADVQQWL	22107.56	27505.67	19468.3	14149.35
**NY-ESO-1**	SLLMWITQV	4.55	4405.69	4585.84	377.43

Supplementary Table 6. Summary of the T_m (ºC) calculations for HLA-B*08:01-A*02:01 refolded with the CMV, EBV* (EBV* = FLRGRAJGL, where J is the 3-amino-3-(2-nitrophenyl)-propionic acid) and the UV-mediated peptide exchange experiments.

Exchanged Peptide	T_m (ºC)
**CMV**	49.8 ± 0.3
**EBV***	48.2 ± 0.1
**EBV**	52.9 ± 0.3
**p29**	40.4 ± 0.1
**p29^{N5R}**	63.6 ± 0.2
**p90**	37.9 ± 0.1
**TAX9**	N/A**
**B40**	N/A**

****N/A, no exchange.**

References

1. Thomsen MCF, Nielsen M. Seq2Logo: a method for construction and visualization of amino acid binding motifs and sequence profiles including sequence weighting, pseudo counts and two-sided representation of amino acid enrichment and depletion. *Nucleic Acids Res* (2012) 40:W281-287. doi: 10.1093/nar/gks469
2. Sievers F, Wilm A, Dineen D, Gibson TJ, Karplus K, Li W, Lopez R, McWilliam H, Remmert M, Söding J, et al. Fast, scalable generation of high-quality protein multiple sequence alignments using Clustal Omega. *Mol Syst Biol* (2011) 7:539. doi: 10.1038/msb.2011.75
3. Robert X, Gouet P. Deciphering key features in protein structures with the new ENDscript server. *Nucleic Acids Res* (2014) 42:W320-324. doi: 10.1093/nar/gku316
4. Kumar S, Stecher G, Tamura K. MEGA7: Molecular Evolutionary Genetics Analysis Version 7.0 for Bigger Datasets. *Molecular Biology and Evolution* (2016) 33:1870–1874. doi: 10.1093/molbev/msw054
5. Letunic I, Bork P. Interactive Tree Of Life (iTOL) v5: an online tool for phylogenetic tree display and annotation. *Nucleic Acids Research* (2021) 49:W293–W296. doi: 10.1093/nar/gkab301
6. Borrman T, Cimons J, Cosiano M, Purcaro M, Pierce BG, Baker BM, Weng Z. ATLAS: A database linking binding affinities with structures for wild-type and mutant TCR-pMHC complexes: Linking TCR-pMHC Affinities with Structure. *Proteins* (2017) 85:908–916. doi: 10.1002/prot.25260
7. Robinson J. IMGT/HLA and IMGT/MHC: sequence databases for the study of the major histocompatibility complex. *Nucleic Acids Research* (2003) 31:311–314. doi: 10.1093/nar/gkg070
8. Leman JK, Weitzner BD, Lewis SM, Adolf-Bryfogle J, Alam N, Alford RF, Aprahamian M, Baker D, Barlow KA, Barth P, et al. Macromolecular modeling and design in Rosetta: recent methods and frameworks. *Nat Methods* (2020) 17:665–680. doi: 10.1038/s41592-020-0848-2
9. Reynisson B, Alvarez B, Paul S, Peters B, Nielsen M. NetMHCpan-4.1 and NetMHCIIpan-4.0: improved predictions of MHC antigen presentation by concurrent motif deconvolution and integration of MS MHC eluted ligand data. *Nucleic Acids Res* (2020) 48:W449–W454. doi: 10.1093/nar/gkaa379

Appendix I. *Rosetta* scripts used in this study.

Script 1. Preprocessing of a sample PDB file (PDB_ID.pdb)

```
$python pdbtools/pdb_delres.py -181: PDB.pdb > PDB_trim.pdb
$python pdb-tools-master/pdbtools/pdb_delhetatm.py PDB_trim.pdb > PDB_trim_noHet.pdb
$python pdb-tools-master/pdbtools/pdb_delchain.py -B PDB_trim_noHet.pdb >
PDB_trim_noHet_noB.pdb
```

Script 2. Pymol script for identifying the peptide binding groove

```
def get_groove(pdb, distance = 5, peptide_chain = "B"):

	"""finds all groove residues within distance from peptide
	returns a list of residue numbers"""

	res_in_grv = []
	cmd.select("groove", "(br. sc. within {0} of ({1}_{2})) and {1}_A".format(distance, pdb,
peptide_chain))
	selection = "groove"
	counter = 0
	extra = ""
#gets list of residues in an object
	objs=cmd.get_object_list("groove")
	m1=cmd.get_model("groove")
	for x in range(len(m1.atom)):
		if m1.atom[x-1].resi!=m1.atom[x].resi:
		res_in_grv.append(int(m1.atom[x].resi))
		counter+=1
	print ("Residues in '%s%s': %s" % (selection, extra, counter))
return res_in_grv
```

Script 3. Threading a protein sequence through a protein structure

```
Rosetta/main/source/bin/partial_thread.macosclangrelease \
    -database Rosetta/main/database \
    -in:file:fasta chimera.fasta \
    -in:file:alignment 5hhn_on_4qrt_trim_noHet_noB.aln \
    -in:file:template_pdb PDB_trim_noHet_noB.pdb \
    -ignore_unrecognized_res
```

Script 4. Command for relaxing a protein structure and calculating total and binding energies

```
Rosetta/main/source/bin/rosetta_scripts.default.macosclangrelease -s chimera.pdb -suffix _relaxed_repacked_sep -no_optH false -use_input_sc -nstruct 3 -parser:protocol relax.xml -flip_HNQ -ignore_zero_occupancy false > script_output.out
```

Script 5. Rosetta scripts relax.xml file

```
<ROSETTASCRIPTS>
  <SCOREFXNS>
    <ScoreFunction name="r15" weights="ref2015" />
  </SCOREFXNS>
  <RESIDUE_SELECTORS>
    #anchor positions
    <Index name="anchors" resnums="1B,2B,3B,4B,5B,6B,7B,8B,9B" />
  </RESIDUE_SELECTORS>
  <PACKER_PALETTES>
  </PACKER_PALETTES>
  <TASKOPERATIONS>
    <InitializeFromCommandline name="init"/>
    <IncludeCurrent name="inclcur"/>
    #dont use specific rotamers of aromatic residues, recommended as they are almost never
incorporated
    <LimitAromaChi2 name="limitchi2" />
    <DisallowIfNonnative name="only_native_C" disallow_aas="C"/>
    <OperateOnResidueSubset name="constant_anchors" selector="peptide" >
      <PreventRepackingRLT/>
    </OperateOnResidueSubset>
    <ExtraRotamersGeneric name="extrachi" ex1="1" ex2="1" ex1_sample_level="1"
ex2_sample_level="1" />
  </TASKOPERATIONS>
  <MOVE_MAP_FACTORIES>
    <MoveMapFactory name="movemap_peptide" bb="1" chi="1">
        <Backbone enable="0" residue_selector="anchors" />
        <Chi enable="0" residue_selector="anchors" />
```

```
        </MoveMapFactory>
    </MOVE_MAP_FACTORIES>
    <SIMPLE_METRICS>
    </SIMPLE_METRICS>
    <FILTERS>
    </FILTERS>
    <MOVERS>
        <FastRelax name="post_design_relax"
            scorefxn="r15"
            disable_design="true"
            task_operations="init,inclcur,limitchi2,only_native_C,extrachi,constant_anchors" repeats="3"
                delete_virtual_residues_after_FastRelax="true"
            movemap_factory="movemap_peptide"/>
        <InterfaceAnalyzerMover name="interface_energy" scorefxn="r15" pack_separated="true"
pack_input="false" resfile="false" use_jobname="true" />
    </MOVERS>
    <PROTOCOLS>
        <Add mover="post_design_relax" />
        <Add mover="interface_energy" />
    </PROTOCOLS>
    <OUTPUT scorefxn="r15" />
</ROSETTASCRIPTS>
```

Appendix II. List of abbreviations used in this study.

**Abbreviation**	**Definition**
ATLAS databse	Accounting Transaction Ledger Archival System database
β_2m	β_2-microglobulin
BSP	BirA Substrate Peptide
CARs	Chimeric Antigen Receptors
CD8+ T cells	Cytotoxic T cell
CDRs	Complementarity-Determining Regions
CMV	Cytomegalovirus
DSF	Differential Scanning Fluorimetry
EBV	Eppstein-Barr virus
ER	Endoplasmic Reticulum
HIV	Human Immunodeficiency virus
HLAs	Human Leucocyte Antigens
IMGT/HLA database	ImMunoGeneTics project/HLA database
MHC-I	Major Histocompatibility Complex class I
PB positions	Peptide-only binding positions
PDB	Protein Data Bank
PDB ID	Protein Data Bank Identification number
pHLA	peptide-HLA complex
pMHC-I	peptide-MHC-I complex
PTB positions	peptide-TCR-binding positions
RMSD	Root Mean Square Deviation
scFv	Single-Chain Fragment Variable
SEC	Size-Exclusion Chromatography
SPR	Surface Plasmon Resonance
TB positions	TCR-only binding positions
TCRs	T cell receptors
UV	Ultraviolet radiation
